# The EDGE2 protocol: Advancing the prioritisation of Evolutionarily Distinct and Globally Endangered species for practical conservation action

**DOI:** 10.1371/journal.pbio.3001991

**Published:** 2023-02-28

**Authors:** Rikki Gumbs, Claudia L. Gray, Monika Böhm, Ian J. Burfield, Olivia R. Couchman, Daniel P. Faith, Félix Forest, Michael Hoffmann, Nick J. B. Isaac, Walter Jetz, Georgina M. Mace, Arne O. Mooers, Kamran Safi, Oenone Scott, Mike Steel, Caroline M. Tucker, William D. Pearse, Nisha R. Owen, James Rosindell

**Affiliations:** 1 Conservation and Policy, Zoological Society of London, Regent’s Park, London, United Kingdom; 2 Department of Life Sciences, Silwood Park Campus, Imperial College London, Ascot, Berkshire, United Kingdom; 3 IUCN SSC Phylogenetic Diversity Task Force, London, United Kingdom; 4 Science and Solutions for a Changing Planet DTP, Grantham Institute, Imperial College London, South Kensington, London, United Kingdom; 5 Institute of Zoology, Zoological Society of London, Regent’s Park, London, United Kingdom; 6 Global Center for Species Survival, Indianapolis Zoological Society, Indianapolis, Indiana, United States of America; 7 BirdLife International, David Attenborough Building, Cambridge, United Kingdom; 8 School of Philosophical and Historical Inquiry, The University of Sydney, Sydney, Australia; 9 Royal Botanic Gardens, Kew, Richmond, Surrey, United Kingdom; 10 UK Centre for Ecology & Hydrology, Crowmarsh Gifford, Wallingford, United Kingdom; 11 Department of Ecology and Evolutionary Biology, Yale University, New Haven, Connecticut, United States of America; 12 Center for Biodiversity and Global Change, Yale University, New Haven, Connecticut, United States of America; 13 Department of Genetics, Evolution & Environment, University College London, London, United Kingdom; 14 Biological Sciences, Simon Fraser University, Burnaby, BC, Canada; 15 Max-Planck Institute of Animal Behavior, Department of Migration, Radolfzell, Germany; 16 University of Konstanz, Department of Biology, Konstanz, Germany; 17 School of Life Sciences, University of Essex, Colchester, United Kingdom; 18 Biomathematics Research Centre, University of Canterbury, Christchurch, New Zealand; 19 Environment, Ecology and Energy Program, University of North Carolina at Chapel Hill, Chapel Hill, North Carolina, United States of America; 20 Department of Biology and Ecology Center, Utah State University, Logan, Utah, United States of America; 21 On the EDGE Conservation, London, United Kingdom; NCBS: National Centre for Biological Sciences, INDIA

## Abstract

The conservation of evolutionary history has been linked to increased benefits for humanity and can be captured by phylogenetic diversity (PD). The Evolutionarily Distinct and Globally Endangered (EDGE) metric has, since 2007, been used to prioritise threatened species for practical conservation that embody large amounts of evolutionary history. While there have been important research advances since 2007, they have not been adopted in practice because of a lack of consensus in the conservation community. Here, building from an interdisciplinary workshop to update the existing EDGE approach, we present an “EDGE2” protocol that draws on a decade of research and innovation to develop an improved, consistent methodology for prioritising species conservation efforts. Key advances include methods for dealing with uncertainty and accounting for the extinction risk of closely related species. We describe EDGE2 in terms of distinct components to facilitate future revisions to its constituent parts without needing to reconsider the whole. We illustrate EDGE2 by applying it to the world’s mammals. As we approach a crossroads for global biodiversity policy, this Consensus View shows how collaboration between academic and applied conservation biologists can guide effective and practical priority-setting to conserve biodiversity.

## Abstract 2

The Evolutionarily Distinct and Globally Endangered (EDGE) metric is used to prioritize threatened species for practical conservation. This Consensus View presents EDGE2, a protocol that draws on a decade of research and innovation to develop an improved, consistent methodology for prioritizing species conservation efforts.

## Introduction

### Why conserve evolutionary history?

Human actions are threatening global biodiversity at unprecedented rates [1]. Declines in biodiversity imperil nature and its capacity to provide benefits to society [2]. There are, however, different currencies by which biodiversity is measured and prioritised, one of which is evolutionary history, the amount of which contained in a set of taxa can be measured using phylogenetic diversity (PD) [3]. The PD of a set of taxa is calculated by summing the phylogenetic branch lengths spanning the set, typically on a dated phylogenetic tree.

While evolutionary history arguably reflects a fundamental component of biodiversity with its own intrinsic value [4–7], the motivations for its conservation go beyond this. Evolutionary history can be linked to the conservation of feature diversity (the different evolutionary features of species), and so to future options for humanity (or “biodiversity option value”; [3,8]). Therefore, by preserving PD, we expect to preserve its associated diversity of features and, thus, maintain the benefits and future options these features contribute to humanity [9–12]. Further, conservation initiatives that incorporate evolutionary history have the potential to capture other desirable components of biodiversity (e.g., “functional” trait diversity; [13–16]).

The importance of halting the loss of evolutionary history has been recognised by the Members of the International Union for Conservation of Nature (IUCN), who adopted Resolution WCC-2012-Res-019-EN, which calls for more “conservation initiatives that target species, especially those of high evolutionary significance” [17]. In light of this, the IUCN has now established a Phylogenetic Diversity Task Force to provide expertise on the inclusion of PD in conservation strategies for practitioners, decision-makers, and the public [18]. Further, the Intergovernmental Science-Policy Platform for Biodiversity and Ecosystem Services (IPBES) has adopted PD as an indicator of the overall capacity of biodiversity to support a good quality of life into the future (the “maintenance of options”; [10]). A PD indicator, along with an index tracking the conservation of the most evolutionarily distinct and threatened species (the “EDGE Index”; [19]), have also been included as indicators for the United Nations Convention on Biological Diversity’s (CBD) draft post-2020 Global Biodiversity Framework (GBF) [20].

### The history of the EDGE approach

In 2007, the Zoological Society of London (ZSL) established the “EDGE” approach as a method for identifying species that should be prioritised for the conservation of threatened evolutionary history. This was underpinned by a metric combining a measure of evolutionary distinctiveness (ED) with values for species’ risk of extinction (global endangerment (GE)) to calculate “EDGE scores” and thereby generating priority rankings [21], or “EDGE Lists.”

ED assigns each species a “fair proportion” of the total PD. To do this, the PD (length) of each phylogenetic branch is divided equally among all living descendants [22]. Species with long ancestral branches that are shared with relatively few other species are therefore responsible for greater amounts of PD than species with short ancestral branches that are shared among much larger groups of descendants. The ED of species *i* can be written as:

$${ED}_{i}=\sum_{j=1}^{n_{i}}\frac{L_{i,j}}{N_{i,j}}$$

Here, *L*_*i*,1_ gives the terminal branch length (TBL) of species *i*, *L*_*i*,*j*_ for 2≤*j*≤*n*_*i*_ gives the length of all internal branches that are ancestral to species *i*, and *N*_*i*,*j*_ gives the total number of descendants of each of these same branches. ED is calculated on a dated phylogeny to provide values measured in millions of years.

GE utilised weightings of extinction risk derived from the categories of the IUCN Red List of Threatened Species that are already widely used to produce Red List Indices [23]. Following Isaac and colleagues [21], the GE of species *i* (hereafter *GE*_*i*_) was given as 0 where species *i* is “Least Concern” (LC), 1 where it is “Near Threatened” (NT), 2 where it is “Vulnerable” (VU), 3 where it is “Endangered” (EN), and 4 where it is “Critically Endangered” (CR). Hereafter, we refer collectively to these as “data-sufficient” categories. In the original metric, Extinct in the Wild species were not included. The EDGE score for any species *i* was then calculated using the EDGE metric as:

$${EDGE}_{i}=\ln\left(1+{ED}_{i}\right)+{GE}_{i}\times ln(2)$$

Following Isaac and colleagues [21], species in threatened IUCN Red List categories of VU, EN, or CR (collectively referred to as threatened) with above-median ED for their clade were identified as priority “EDGE Species,” with particular conservation attention given to the highest-ranking 100, 50, or 25 species in particular clades [24,25].

This approach has been used to generate priority EDGE Lists for mammals [21,26], amphibians [27], birds [28], corals [29], reptiles [24], gymnosperms [30], and sharks and rays [31]. http://www.edgeofexistence.org/The EDGE approach has informed direct conservation action for many of the EDGE Species highlighted and has served as the basis for conservation efforts of ZSL’s own EDGE of Existence programme that has supported over 120 conservation projects worldwide on priority EDGE Species.

ED scores have often been utilised as a measure of species distinctiveness [31–34], and EDGE Species are increasingly recognised as being of global conservation importance [17,35,36]. EDGE data underpin initial estimations by IPBES for their PD indicator [10,37,38], which approximates expected loss of PD [39]. EDGE data also inform conservation grant mechanisms, such as the IUCN Species Survival Commission (SSC) EDGE Internal Grant [40], which funds Red Listing and action planning for evolutionarily distinct species and lineages, and the conservation needs assessments utilised by Amphibian Ark’s conservation grants programme [41].

### Revisiting the EDGE approach

The uptake of PD-informed approaches in research, policy, and applied conservation has occurred alongside great research advances across the field of phylogenetically informed conservation prioritisation [28,42–46]. The original EDGE approach, while having strong theoretical underpinnings, is based on a heuristic metric designed to facilitate swift judgements on prioritising species for conservation according to the information derived from two elements: the ED (a metric of irreplaceability), and the GE (vulnerability) of species. More recent advances in the field of PD conservation have opened new avenues to produce an updated EDGE protocol underpinned by a metric that retains the same elements of irreplaceability (from a phylogenetic perspective) and vulnerability. Key among these has been the quantification of extinction risk [44,47], the incorporation of uncertainty in both phylogeny [24,28,48] and extinction risk [49,50], and the concept of phylogenetic complementarity between species [42,43,51]. Each of these developments indicates a potential weakness in the original EDGE approach and a corresponding opportunity for improvement.

While research advances have provided EDGE lists for various taxonomic groups that are used in practical conservation [24,28,30,31,52–54], these are all based on the original EDGE approach. However, conceptual advances to the phylogenetically informed prioritisation of species, suggested by other research [42,45,55,56], have not become part of conservation practice. Arguably, this is because the successful uptake and application of the original EDGE metric lessened the impetus to overhaul the approach for applied work. Consequently, a high level of confidence is required by conservation practitioners to justify changes to existing practice that would result in changes in winners and losers in terms of species receiving conservation attention. To date, conceptual advances have not been mutually exclusive and have not come packaged with a complete set of standardised procedures that would produce actionable results.

To address these issues, we convened a workshop in April 2017 to assess whether and how to update the original EDGE approach in light of a decade of research advances since 2007. It was essential to carefully assess and justify any changes, so we brought together a wide variety of experts and required everyone to be in agreement on all decisions made so as to produce a credible, comprehensive, and progressive update. Consensus was tested throughout the process by voting with tweaks being made and caveats added to address any concerns raised. In this manuscript, we describe the updated protocol that we refer to as EDGE2. We characterise the key advantages of the EDGE2 protocol as a tool to prioritise the conservation of threatened evolutionary history, and, finally, we provide guidance for interpreting EDGE2, illustrated with an application to the world’s mammals.

## Introducing the EDGE2 protocol

To ensure its effective and continued use to guide conservation practice, we developed a comprehensive protocol for EDGE2. This includes an EDGE2 metric but also includes the other components necessary to deal with uncertainty in extinction risk data, uncertainty in phylogenetic data, and production of final priority lists. While the objective is to provide a full protocol for consistency of future work that builds EDGE lists or uses them in downstream analyses, we identify some components of the protocol that can naturally be carved out as areas for independent ongoing development. Future research may vary such individual components while keeping everything else the same. The conceptual separation of components should also make it easier for a future updated consensus view to change individual components without needing to revisit the entire protocol.

### The EDGE2 metric

The EDGE2 metric component is based on earlier probabilistic approaches to phylogenetically informed conservation prioritisation [42,43,57,58] that measure the avertable loss of PD through the conservation of individual species. The EDGE2 metric is mathematically equivalent to the “Heightened EDGE” (HEDGE) metric where conservation of a species makes its future secure (theorem 4.1(i) in Steel and colleagues [42]). We frame the EDGE2 metric as the product between two familiar terms: an ED component (“ED2”; the irreplaceability of a species) and an extinction risk component (“GE2”; the vulnerability of a species), which follows earlier formulations to calculate species-specific expected losses of evolutionary history [59]. Thus, the EDGE2 score of species *i* can be given as:

$${EDGE2}_{i}={ED2}_{i}\times {GE2}_{i}$$

### GE2

In the original formulation of EDGE, the metric dictates that, should the extinction risk of all species be equal, the most evolutionarily distinct species will be prioritised [21]. However, given that extinction risk varies across species, the original formulation of GE was designed to weight the ED of species so that high ED species would not be ignored in the presence of the many species that are more threatened, but less distinct.

We regard GE2_*i*_ as a GE weighting, now between 0 and 1, for species *i* relative to other species. In order for the EDGE2 protocol to be standardised, we set GE2_*i*_ = *p*_*i*_, the probability of extinction for species *i* at some given future time. The EDGE2 protocol could be straightforwardly adapted to incorporate other quantifications of extinction risk for assessing different conservation goals. For example, if one was to evaluate the potential impacts of particular conservation actions, GE2_*i*_ may be replaced by a measure to estimate the likely reduction in probability of extinction from a particular action [43,45,56]. Setting GE2_*i*_ = *p*_*i*_ should be interpreted as taking a particular conservation action, which means species *i* is protected from extinction instead of going extinct with probability *p*_*i*_ (following [42]; theorem 4.1(i)).

### Quantifying probabilities of extinction with uncertainty

There is limited consensus in the literature about how to quantify probabilities of extinction [44,47,60,61]. In the spirit that the original GE scores are arbitrary, and something had to be agreed in order to proceed, we convert IUCN Red List categories to extinction probabilities based on the 50-year time horizon specified in Mooers and colleagues [44] (a study notable for examining the impact of time-horizon and *p* definition on conservation prioritisation), with CR mapped to an extinction risk weighting of 0.97. In the absence of any agreed alternative, we determined the remaining Red List categories by retaining the approach from the original EDGE metric, which approximated the expected loss of evolutionary history when extinction risk halves with each step down in extinction risk severity [21]. Thus, EN species therefore receive a weighting of 0.485, VU = 0.2425, NT = 0.12125, and LC = 0.060625 (for details, see “values of extinction risk” in S1 Text). Future calculations of EDGE2 should adhere to consistent values of extinction risk to ensure comparability with any existing EDGE2 prioritisations.

**Fig 1:**
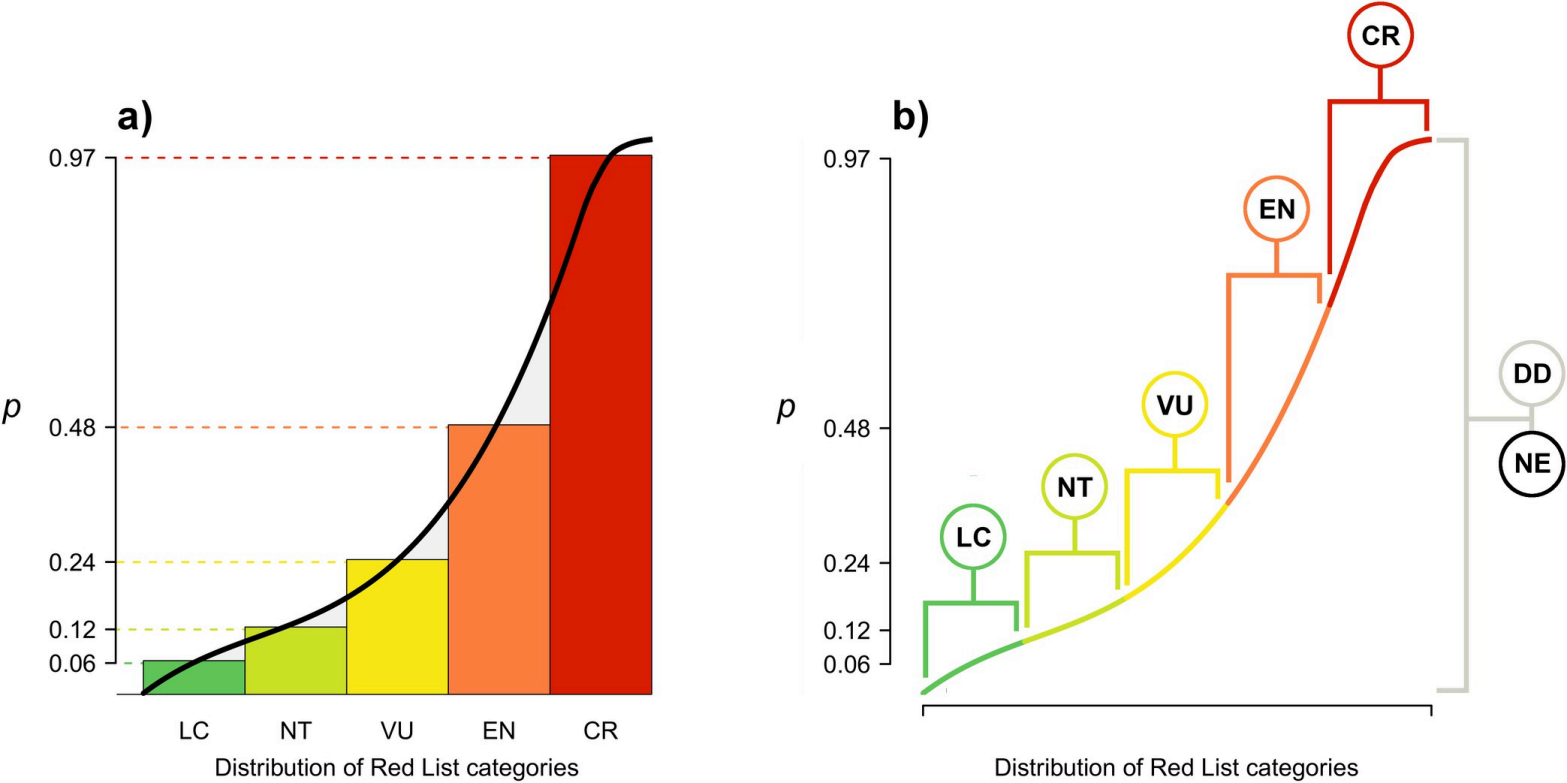
Generating a distribution of probabilities of extinction (*p*) from discrete IUCN Red List categories. To generate a continuous distribution of values from which to draw *p*, we (**a**) created a rank plot of ranked extinction risk (x-axis) assigned to the designated median *p* for each IUCN Red List category (y-axis; see “Quantifying probabilities of extinction with uncertainty” section). We fitted a quartic curve through the five median values for each Red List category and bounded the resulting curve to return values between 0.0001 and 0.9999 (not wishing to ever return a 0 or 1 extreme)—see methods. From this curve, we could extract a set of values associated with each Red List category (**b**), the median of which was equal to the predefined *p* for each category (CR = 0.97; EN = 0.485; VU = 0.2425; NT = 0.12125; LC = 0.060625). The *p* for DD and unassessed (NE) species is drawn from the entire distribution to capture uncertainty around their extinction risk. The data underlying this Figure can be found in S2 Data. CR, Critically Endangered; DD, Data Deficient; EN, Endangered; IUCN, International Union for Conservation of Nature; LC, Least Concern; NE, Not Evaluated; NT, Near Threatened; VU, Vulnerable.

We incorporated uncertainty in the quantification of extinction risk based on the idea that if we were to rank species by their true probability of extinction, the probabilities will change smoothly reflecting biological processes, and not jump as we move between the discrete human-inferred Red List categories (Fig 1). To replicate this, we generated a distribution of GE2 scores by fitting a quartic curve through the five median values for each Red List category and bounded the resulting curve to return values between 0.0001 and 0.9999 (not wishing to ever return a 0 or 1 extreme). From this curve, we could extract a set of values associated with each Red List category, the median of which was equal to the predefined GE2 score for each category (see above; Fig 1). This approach then allows us to assign GE2 scores for all species, including Data Deficient (DD) and unassessed (Not Evaluated (NE)) species, which are then drawn a number of times (100 < *n* ≤ 1,000 to adequately incorporate uncertainty into EDGE calculations) from the entire distribution to capture uncertainty around both their extinction risk and the resulting variation in EDGE2 scores (for further information, see “incorporating GE2 uncertainty” in S1 Text).

### ED2

One attractive element of the original EDGE formulation is that individual species were assigned a measure of distinctiveness (irreplaceability) based on their phylogenetic position and relationships: their ED [21]. We now advance the EDGE metric to incorporate PD complementarity into the ED component. This means that when calculating ED2 for a species, we consider the extinction risk of its close relatives to better reflect the expected contribution of the species to PD in the future [42,43,62]. Thus, a species with threatened close relatives will have a greater responsibility for shared internal phylogenetic branches compared with a species with close relatives of low conservation concern (see “PD complementarity” in S1 Text).

ED2 is conceptually a special case of “expected distinctiveness” [58] and mathematically equivalent to “heightened evolutionary distinctiveness” (HED) [42]. We express ED2 for species *i* as follows:

$${ED2}_{i}={TBL}_{i}+\sum_{j=2}^{n_{i}}\left(L_{i,j}\times \prod_{k\in C_{i,j}-\{i\}}p_{k}\right)$$

where ED2_*i*_ comprises TBL_*i*_ (TBL of species *i*) plus another component. This is intuitive as the PD captured by the TBL is not shared with any other species while the other component captures shared contributions. The same is true of original ED, but here, we write it explicitly. The set *C*_*i*,*j*_ represents all species descended from the corresponding branch with length *L*_*i*,*j*_ (as defined earlier), and *p*_*k*_ is the probability of extinction of species *k* [39]. ED2_*i*_ is therefore species *i*’s expected unique PD (TBL) at some point in the future, assuming all other species in the tree survive or perish as a function of their probabilities of extinction (product of all *p*_*k*_). When species *i* has few close relatives, all with high *p*, it is expected to contribute a larger amount of unique PD than that of its current TBL, because it is more likely that all its close relatives will perish. In contrast, a species with many secure close relatives is not expected to contribute much more than its current TBL. A change in *p* for one species therefore changes the ED2 scores of the species related to it. The formula for ED2_*i*_ considers each branch corresponding to *L*_*i*,*j*_ in turn and calculates its probability of becoming part of the terminal branch for species *i* in the future due to the extinction of all other descendants. So that the EDGE2 scores of individual species are not sensitive to the clade being analysed, it is necessary to consider every ancestral branch to the stem, until such a time that either the origin of life has been reached, or the change to the score from going any further can be shown to be negligible compared to the precision of the scores themselves. The latter will be reached rapidly as branches with multiple nonthreatened descendants are incorporated, given the expected PD loss is calculated by multiplying the branch length by the product of the GE2 of all descendant species.

### Quantifying ED2 with uncertainty

Since the inception of the EDGE metric, it has been the standard to incorporate all described species to ensure all species of conservation concern are included where possible, particularly those species from old lineages with few, if any, closely related species [21,24,26,27]. The original EDGE approach had a set of rules for resolving uncertainty in ED scores arising from lack of phylogenetic structure (polytomies) in poorly known regions of the phylogeny—based on assumptions regarding net diversification rates—and also from the omission of species from the phylogeny, based on the observed ED scores of their closest relatives [21].

It is increasingly common for fully resolved phylogenetic trees to be generated that include all described species of large clades (at that time), including those species for which we have no genetic data [30,52,53,63–66]. Such synthetic phylogenetic trees often utilise taxonomic information and established phylogenetic imputation methods (e.g., [67]) to generate what are essentially Bayesian posterior distributions of imputed phylogenetic trees [68] (though other approaches, such as source-tree bootstrapping, are also available; [69]). This approach is inherently uncertain (more so than other phylogenetic hypotheses), particularly in regard to the phylogenetic structure of regions of the phylogenies with large proportions of imputed species, and this uncertainty must be reflected in the calculation of ED2 scores and the subsequent identification of conservation priorities [24,48]. ED2 is therefore calculated across a large distribution of imputed phylogenetic trees (100 < *n* ≤ 1,000 to capture phylogenetic uncertainty; [28,48]) that comprise all described species for the clade being assessed. This generates a Bayesian posterior distribution of scores from which we can determine the robustness of priority EDGE2 species using established criteria [48].

### Advances of the EDGE2 metric

The key advance of the EDGE2 metric from the original EDGE metric is the incorporation of PD complementarity in the ED2 component and the consequent adoption of the probabilistic framework for calculating expected PD losses. This incorporation of PD complementarity into the EDGE metric is widely supported in the literature [42,43,45,56,70]. However, our calculation and conceptualisation of the EDGE2 metric have two significant divergences from the original HEDGE calculation.

First, our explicit deconstruction of the species’ ED2 into the unique terminal and shared internal components of the score allows us to estimate both the minimum unique PD expected to be lost with the extinction of a species and the relative importance of internal branches for a species’ ED2 score. This indicates the degree of responsibility a species has for internal phylogenetic branches and, thus, species whose conservation would safeguard imperilled deep branches of the Tree of Life due to elevated extinction risks of entire genera, families, or even orders (see “terminal versus internal branches” in S1 Text). Second, our distinction between the extinction risk components of the ED2 calculation and the GE2 calculation provides future flexibility in quantifying the impacts of conservation actions. Given that it is unrealistic that a species can ever be completely “secure,” future work may, if suitable data become available, recast GE2 as an expected reduction in probability of extinction within some given timescale. This would make it distinct from the probabilities of extinction used as part of the ED2 calculations, which only give the extinction risk of the species for which they are calculated, not the potential benefit from conservation actions.

### EDGE species and lists

To enable downstream application of the EDGE2 approach, the final EDGE2 score for a species is taken as a measure of the centre of the distribution (the median) of EDGE2 scores generated to account for uncertainty in phylogenetic relationships and extinction risks. Likewise, the final ED2 and GE2 values for a species are also the medians from their respective sampled distributions.

**Fig 2:**
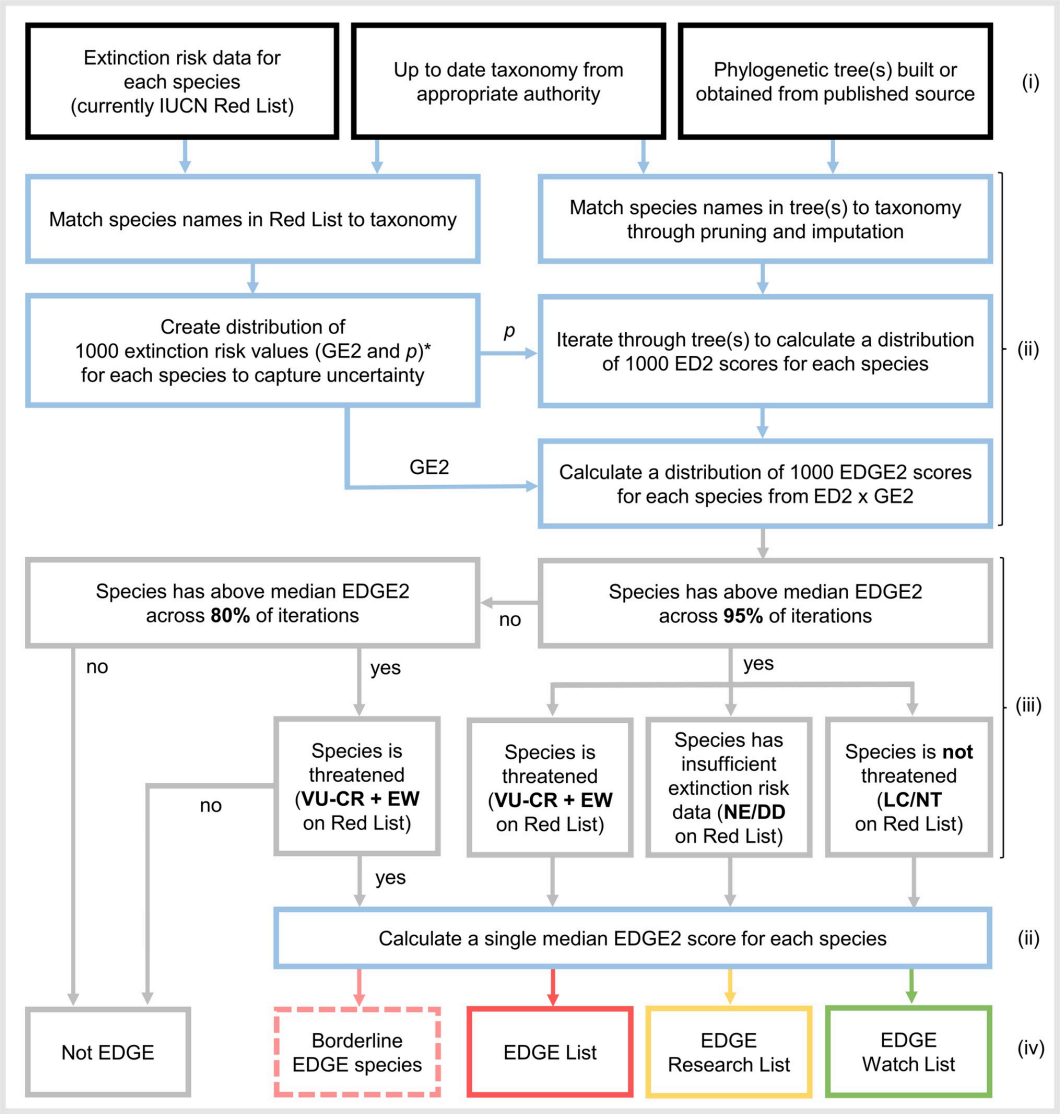
The EDGE2 protocol. The protocol, from data input to final output, for producing a comprehensive EDGE2 prioritisation for a set of species. Stages from top (beginning) to bottom (end): required data (i–input data); data processing stages (ii–blue); selection criteria to be applied (iii–grey); and outputs (iv). *p and GE2 are assigned equivalent values in EDGE2 but may differ in future work given increased understanding of extinction risk, the likely effects of future conservation work, and its distribution across the Tree of Life (see S1 Text). The single median EDGE2 score for each species, based on the values of p and GE2 used in this study, can be compared across studies and clades and should not be sensitive to the size of clade used in the study. In contrast, membership of the EDGE2 list and other related lists is sensitive to the other species included in the study. For example, an EDGE2 mammal should have the same EDGE2 score in a mammal-level study and a tetrapod-level study but might not qualify for the EDGE2 list for all tetrapods if the nonmammalian tetrapods have higher EDGE2 scores, thus reducing the confidence that its EDGE2 score is above the median of the larger group.

In the original EDGE approach, a priority “EDGE Species” was one with an above-median ED score, relative to all species in the clade, which was also threatened (VU, EN, or CR on the Red List) [21,24,26]. EDGE2 follows a similar philosophy: Priority “EDGE2 Species” must be noteworthy both in EDGE2 score and risk of extinction (GE2). We thus maintain the requirement for EDGE2 species to be in a threatened Red List category but also include Extinct in the Wild species in this designation, given their potential for recovery in the wild. We also require the species to score above the median EDGE2 score for all species in the clade across at least 95% of the distribution of scores. This means that we are 95% certain the species has an above-median EDGE2 score after incorporating uncertainty in both extinction risk and phylogeny. Fig 2 summarises how we can use the Red List categories and EDGE2 scores to create lists for practical conservation prioritisation.

We define an EDGE List as comprising all EDGE2 Species (those with EDGE2 values above the median across 95% of iterations and threatened with extinction; Fig 2). We also set criteria to highlight “borderline EDGE Species”; these are species that are threatened with extinction whose certainty around their EDGE score is still relatively high (e.g., above-median EDGE2 >80% of the time) but not enough to meet our threshold of 95% certainty. For taxonomic groups with high phylogenetic uncertainty, these “borderline” species could be included in the EDGE List with a relaxation of the certainty threshold (e.g., from 95% to 80%).

### Recognising other species of importance

We also propose an “EDGE Research List” for species that are credibly above-median EDGE2 but are currently either NE or listed assessed as DD on the IUCN Red List (Fig 2). Given that the GE2 values for DD or NE species under EDGE2 are drawn from all possible values for data-sufficient categories, with a median approximately equivalent to that of VU species [47,71], any EDGE Research species are sufficiently evolutionarily distinct that they would become EDGE species if they were eventually to be assessed (or reassessed for DD species) as VU or above on the IUCN Red List.

The expansion of the EDGE approach to explicitly prioritise species lacking sufficient conservation knowledge for further research extends previous efforts to catalyse targeted extinction risk assessments for highly distinctive species potentially at risk of extinction [27,72]. EDGE Research Lists can inform ongoing efforts to improve our understanding of extinction risk for priority DD species, such as the IUCN SSC’s EDGE internal grants [40] and the Mohamed bin Zayed Species Conservation Fund.

We also define an “EDGE Watch List” for LC and NT species that rank above-median EDGE2 95% of the time despite their low extinction risk and are therefore responsible for securing large amounts of imperilled PD (Fig 2). The maintenance of these species is critical to the persistence of deep branches of the Tree of Life that are currently considered safe.

## EDGE2 real-world application

### Example for the world’s mammals

We applied the EDGE2 protocol to the world’s mammals using a distribution of 1,000 augmented phylogenetic trees from [65] (methods and additional results in S2 Text). We calculated 1,000 values of GE2, ED2, and EDGE2 for 6,253 mammal species, 4,847 of which could be linked to IUCN Red List assessments with a data-sufficient Red List category (for exploration of the impact of phylogenetic imputation on EDGE2, see “imputation analyses” in S2 Text). Median ED2 scores across the 1,000 trees ranged from a minimum of 0.08 MY (*Uroderma bakeri*, Baker’s Tent-making Bat) to a maximum of 77 MY (*Orycteropus afer*, Aardvark), with a median ED2 of 2 MY (S1 Fig). ED2 is strongly positively correlated with TBL (Pearson’s product–moment: r = 0.95, df = 6251, *p* < 0.0001; S2 Fig) and with original ED values calculated following Isaac and colleagues [21] (hereafter ED1; r = 0.829, df = 6251, *p* < 0.0001; S2 Fig; additional results in “comparing measures of ED” in S2 Text).

**Fig 3:**
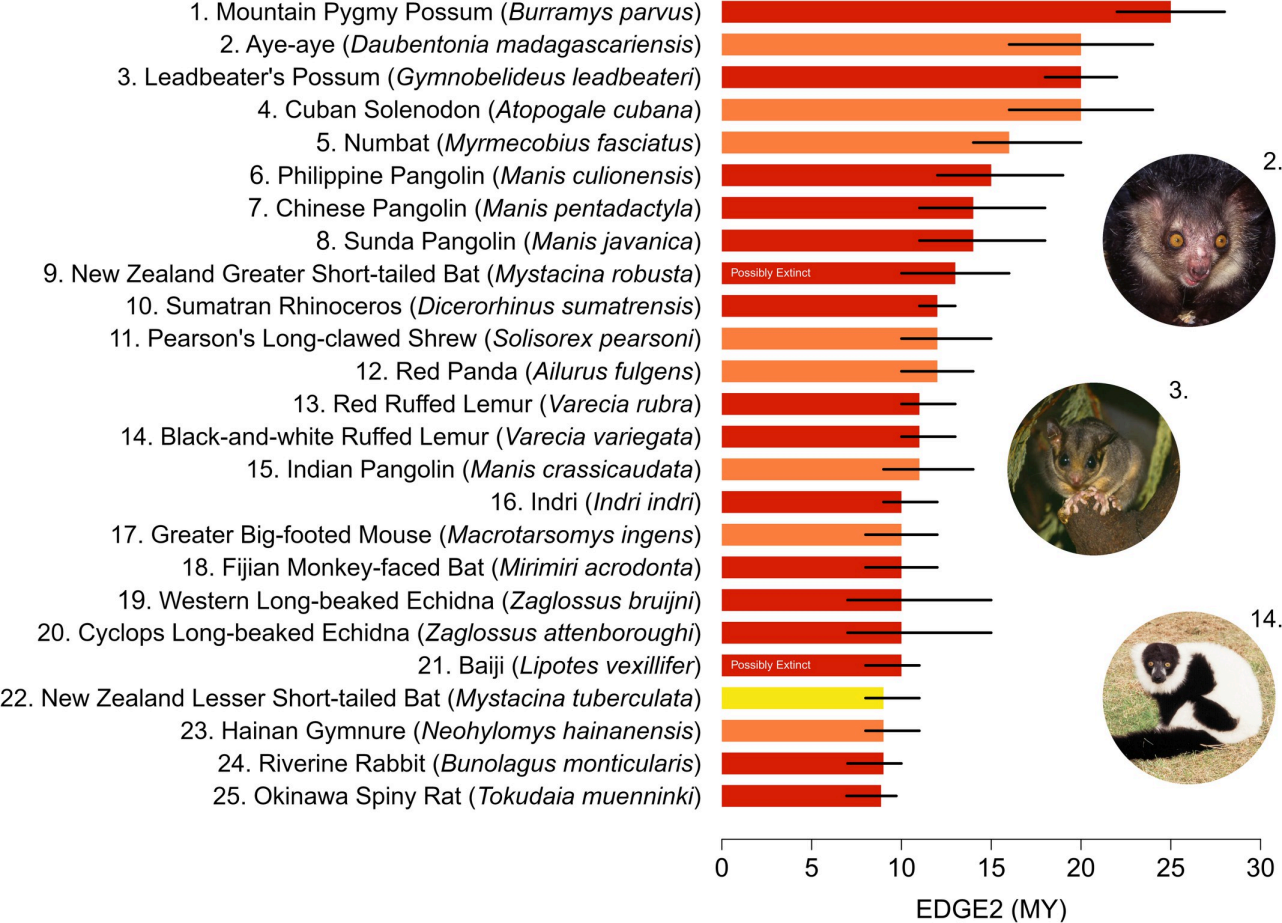
Top 25 EDGE2 Mammal species. The median EDGE2 (bars) and interquartile range of EDGE2 (error bars) for the 25 highest-ranking EDGE2 mammal species. EDGE2 scores calculated across a distribution of 1,000 phylogenetic trees. Colours of the bars represent IUCN Red List categories (red = CR, orange = EN, yellow = VU); “Possibly Extinct” denotes CR species marked with this tag on the Red List. The Full EDGE List is given in the S1 Data. The data underlying this Figure can be found in S2 Data. *Image credits*: *Zoological Society of London*. CR, Critically Endangered; EDGE, Evolutionarily Distinct and Globally Endangered; EN, Endangered; IUCN, International Union for Conservation of Nature; VU, Vulnerable.

The large-headed Capuchin (*Sapajus macrocephalus*) had the lowest EDGE2 score (0.01 MY), and the species with the greatest median EDGE2 score (25 MY of avertable expected PD loss) was the Mountain Pygmy Possum (*Burramys parvus;* Fig 3). The median EDGE2 score for mammals was 0.2 MY of avertable expected PD loss (all EDGE2 scores are available in S1 Data). EDGE2 ranks are strongly positively correlated with EDGE ranks calculated using the original Isaac and colleagues’ [21] approach (hereafter EDGE1) for all extant and data-sufficient mammals (ρ = 0.916, df = 4845, *p* < 0.0001; S3 Fig; additional results in “comparing EDGE priorities” in S2 Text).

We identified 645 “EDGE2 Species” (S1 Data), which together account for 81% of the avertable expected PD loss of all threatened species (1,458 MY). Safeguarding the 100 highest-ranking threatened species (1.6% of mammal species) under EDGE2 would secure 719 MY of PD that would otherwise be lost, which is 23% of avertable expected PD loss across all mammal species (i.e., 23% of summed EDGE2 scores; S4 Fig).

Of the 645 EDGE2 Species identified here, 357 (60%) are also identified as priorities under EDGE1 Species criteria when calculated using the same phylogenetic and extinction risk data. Among EDGE2 Species, higher-priority species have smaller changes in their ranking from EDGE1 to EDGE2 compared with lower-priority species (i.e., high-ranking EDGE2 priorities are more stable, having smaller rank changes between EDGE metrics; ρ = 0.378, df = 626, *p* < 0.001).

The EDGE Research List comprises 15 highly evolutionarily distinct mammal species with limited conservation knowledge (DD or NE on the Red List; S1 Data). Six of the 15 species would be top 100 EDGE2 mammals if they were assessed as VU or higher on the Red List, and the highest-ranking species among these include the long-eared Gymnure (*Hylomys megalotis*; median ED2 = 34 MY), a poorly known relative of hedgehogs, which is thought to resemble some of the earliest mammals and diverged from all other extant mammals more than 30 million years ago, and Owl’s Spiny Rat (*Carterodon sulcidens*; median ED2 = 17 MY), a fossorial relative of the Coypu and hutias.

The EDGE Watch List comprises 82 nonthreatened mammal species (S1 Data). The Watch List includes highly evolutionarily distinct species such as the Aardvark (ED2 = 78 MY) and Duck-billed Platypus (*Ornithorhynchus anatinus*; ED2 = 51 MY). It features mammals that are highly evolutionarily, ecologically, and morphologically distinct, including possibly the world’s smallest mammal, the Bumblebee Bat (*Craseonycteris thonglongyai*) [73]; the smallest extant anteater, the arboreal and nocturnal Silky Anteater (*Cyclopes didactylus*) [74]; and the chronic alcohol-consuming Pen-tailed Treeshrew (*Ptilocercus lowii*) [75]. Of the 82 species on the Watch List, 68 are already NT; it is thus important to recognise them explicitly in the EDGE2 protocol.

### Transitioning from EDGE1 to EDGE2

**Fig 4:**
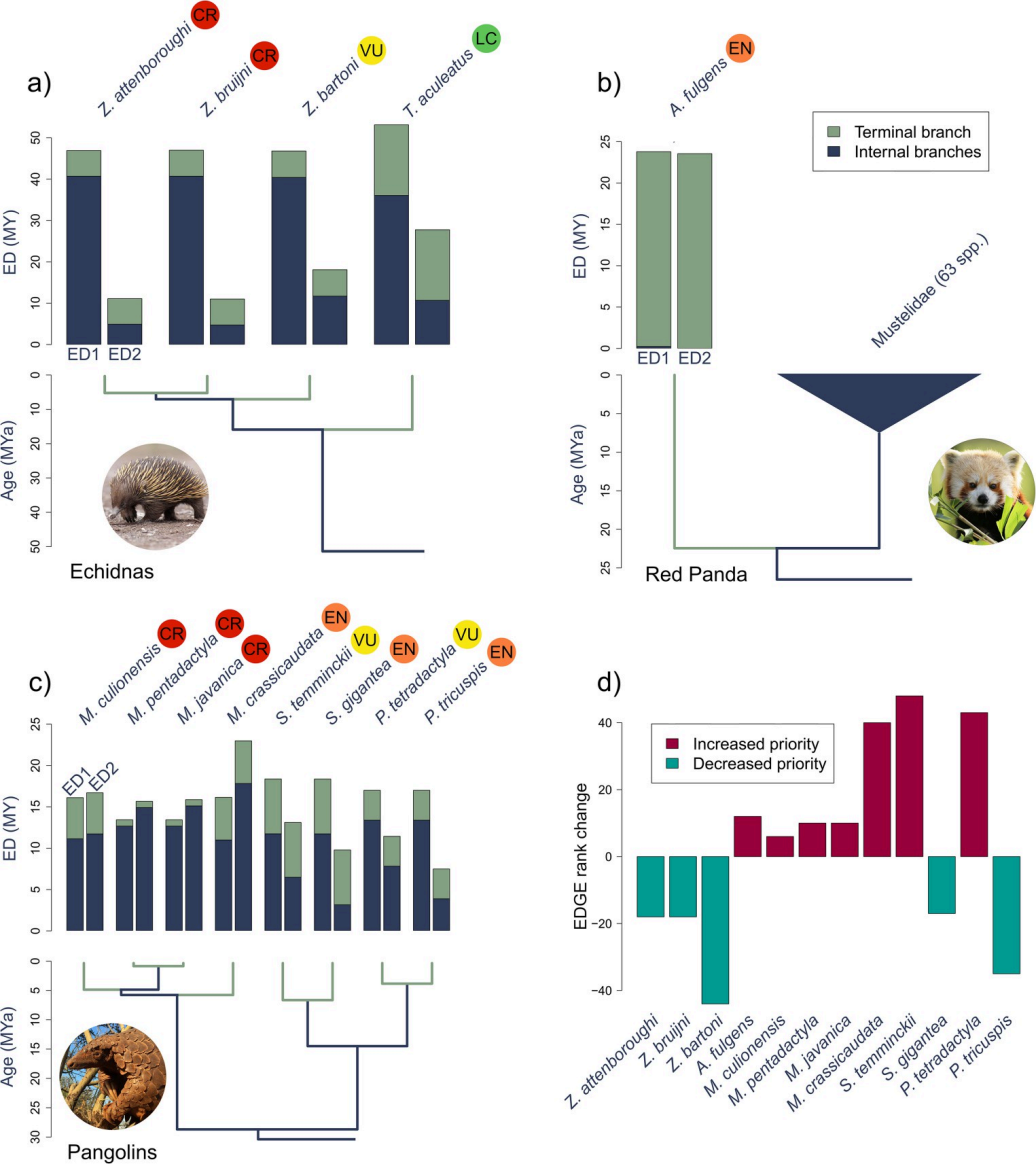
Comparing ED and EDGE scores of priority EDGE species. The change in ED from ED1 to ED2 for (**a**) echidnas (Tachyglossidae); (**b**) the Red Panda (*Ailurus fulgens*); (**c**) pangolins (order Pholidota); and (**d**) the change in priority rank of these species from EDGE1 to EDGE2. The data underlying this Figure can be found in S2 Data. *Image credits*: *echidna*: *Leo Berzins; Red Panda*: *Zoological Society of London; pangolin*: *Wendy Panaino*. CR, Critically Endangered; ED, evolutionary distinctiveness; EDGE, Evolutionarily Distinct and Globally Endangered; EN, Endangered; LC, Least Concern; VU, Vulnerable.

The explicit incorporation of PD complementarity into the EDGE prioritisation has long been advocated for. This, however, leads to different consequences for the ED—and thus the EDGE scores—of species depending on the interplay of evolutionary relationships and extinction risk (Fig 4; and see S5 Fig). For example, the ED of long-beaked echidnas (*Zaglossus* spp.) is reduced under ED2 relative to ED1 because, under original EDGE, the majority of the ED1 score was contributed by interior branches (>85%; Fig 4A). Under EDGE2, the contribution to the ED2 score of internal branches, such as the long internal branch shared with the LC short-beaked Echidna (*Tachyglossus aculeatus*; Fig 4A), is greatly reduced (<50% for *Zaglossus bruijni* and *Zaglossus attenboroughi*) due to the small probability that those branches will be lost. When we consider the Red Panda (*Ailurus fulgens*), there is very little change in its ED from ED1 to ED2 (Fig 4B) as most of its score under both metrics is contributed by its terminal branch, shared with no other species. This is because the most recent internal branch ancestral to the Red Panda is shared with all mustelids (>60 spp.; Fig 4B) and thus contributes very little to ED scores under both EDGE1 and EDGE2.

We see a variety of consequences for pangolins (Pholidota; Fig 4C). The ED score of the EN Indian Pangolin (*Manis crassicaudata*) increases under ED2 relative to ED1 due to the high extinction risk of its three congeners, all of which are CR (Fig 4C). This clustering of high extinction risk means the Indian Pangolin has increased responsibility for the internal branches shared by *Manis* spp. Conversely, the ED of the White-bellied Pangolin (*Phataginus tricuspis*) decreases under ED2 due to the lower extinction risk (relative to *Manis* spp.) of its closest relatives, *Phataginus tetradactyla* and *Smutsia* spp. (Fig 4C). Our deconstruction of ED2 into the unique terminal and shared internal branch components allows us to identify species that are a high priority due to their unique evolutionary history, such as the Red Panda, or their expected responsibility for the persistence of internal branches of the Tree of Life, such as the Indian Pangolin. The conservation of priority species under both scenarios is crucial if we wish to avoid particularly large losses of PD (Fig 4D).

**Fig 5:**
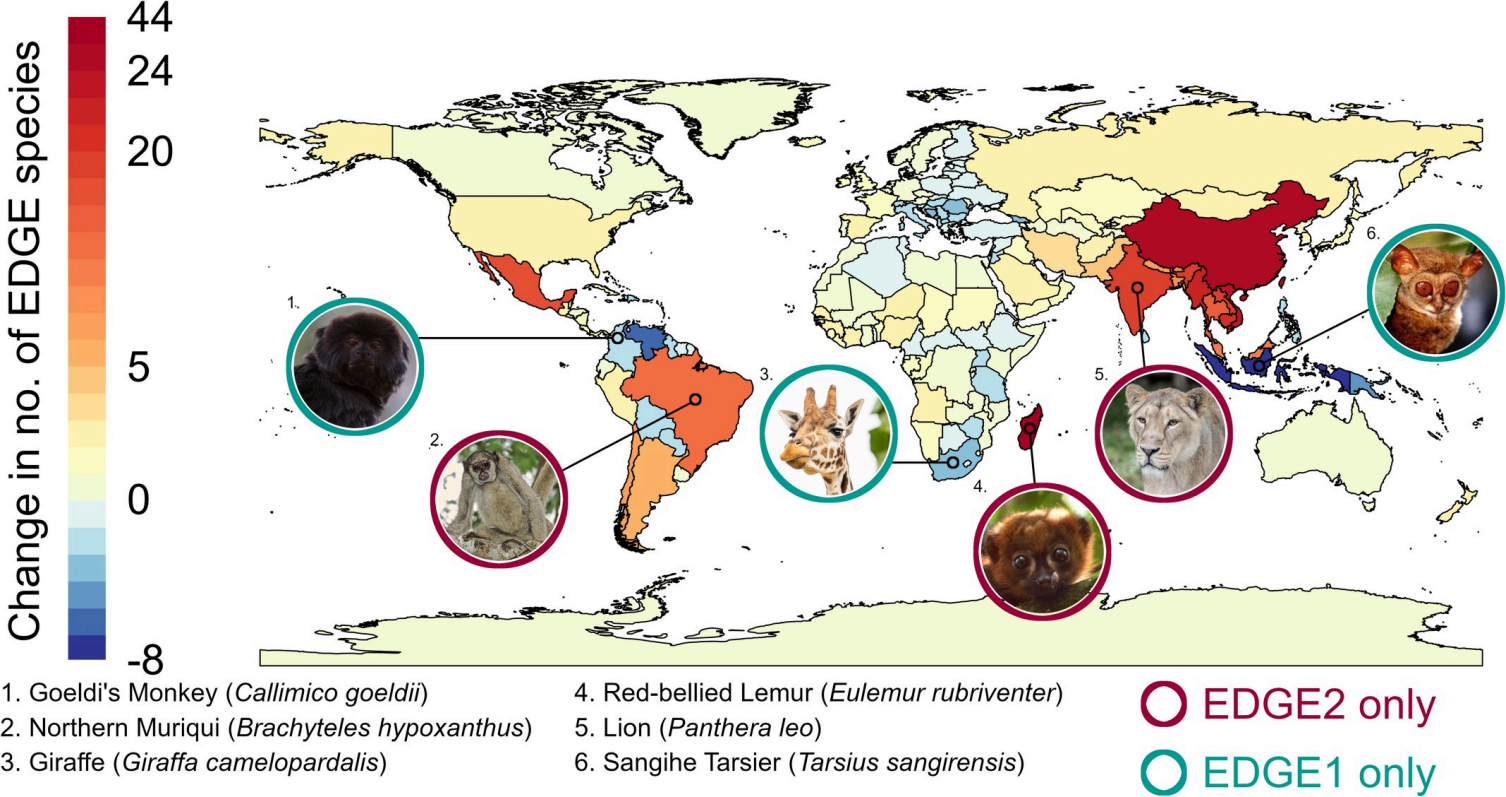
Change in global mammal EDGE species richness. The difference between number of priority EDGE species identified under EDGE1 and EDGE2 at the national level. Positive scores indicate an increase of EDGE species richness under EDGE2, whereas negative scores indicate a decrease from EDGE1. Species featured are either no longer considered EDGE species under the EDGE2 protocol (teal circles) or now considered priority EDGE species under EDGE2 that were not under the EDGE1 approach (maroon circles). Map of country regions from: https://www.naturalearthdata.com/downloads/10m-cultural-vectors/10m-admin-0-countries/. The data underlying this Figure can be found in S2 Data. *Image credits*: *1*: *Glenn van Windt; 2*: *Bill Bouton; 3 and 5*: *Zoological Society of London; 4*: *Rikki Gumbs; 6*: *Myron Shekelle*.

The practical implications of the changes can be visualised spatially when we map EDGE species at the national level for both EDGE1 and EDGE2 (for methods, see “transitioning from EDGE1 to EDGE2” in S2 Text). The transition between metrics leads to gains or losses in numbers of EDGE species in many countries (Fig 5). Though a large proportion of countries remain stable in terms of number of EDGE species (118 of 197 countries vary by fewer than two EDGE species from EDGE1 to EDGE2), there are notable exceptions. The greatest gain of EDGE species is in Madagascar (+44 species), due to the increased priority apportioned to the lemurs, where high extinction risk is clustered on the mammal phylogenetic tree. Conversely, the greatest loss of EDGE species is in Indonesia (−8 species), largely due to the reduced priority of small nocturnal mammals with short (<2 MY) terminal branches and relatives with relatively low extinction risk, such as cuscus, lorises, and tarsiers (Fig 5 and S2 Data). Thus, the change in metric and subsequent criteria for identifying priority EDGE species leads to tangible changes in national responsibilities for the conservation of EDGE species globally, as would also be evidenced in national-level disaggregation of the EDGE Index [76].

Finally, to explore the potential real-world implications of prioritising species for conservation action under EDGE2 compared with EDGE1, we can look to ZSL’s EDGE of Existence programme. The programme has supported conservation projects on 34 EDGE mammal species since 2007, of which 30 remain priority species under EDGE2. However, in its call for funding proposals, the programme states that preference is given to species with higher-priority EDGE ranks. The median rank of supported species under EDGE1 is 55, whereas this significantly increased to 95.5 under EDGE2 (*p* < 0.05; S6 Fig; for methods, see S2 Text). While several other factors influence the allocation of support, from the profile of the applicant and the location of the proposed project to the existing conservation attention on the focal species, this suggests that, while the vast majority of species selected for support would still be eligible for consideration under EDGE2 (89%), their lower ranks under EDGE2 compared with EDGE1 may have reduced their likelihood of selection in favour of higher ranked EDGE2 species (assuming median EDGE rank selection by the programme remained at 55).

### EDGE2 is more than a metric

One key strength of the original EDGE approach was standardisation, with the calculations for ED and values of GE outlined explicitly. This meant that comparable EDGE Lists could be generated for various taxonomic groups from research conducted by different teams of people across a large period of time and facilitated the wider use of ED and EDGE scores for multiple taxa in research [30] and policy reporting [10]. However, previous applications of HEDGE (and related metrics) have varied in their quantification of extinction risk, approach to uncertainty regarding poorly known species, and delineation of priority species, limiting the wider applicability of the research. Here, the EDGE2 protocol is designed to incorporate necessary advances to the EDGE metric while still enabling third parties to generate standardised outputs that can continue to facilitate the wider uptake of phylogenetically informed species conservation.

EDGE2‘s utilisation of uncertainty to set priorities differs from approaches that simply rank species based on a single or median score before applying varying criteria for what is or is not a priority species. For example, Robuchon and colleagues [77] ranked the world’s mammals by their median HEDGE score and designated the species with the highest 25% of scores as their priority species, irrespective of extinction risk. Though Robuchon and colleagues’ use of phylogenetic tree and quantification of extinction risk differs from those used here, we can apply their 25% criterion to our median EDGE2 scores for all mammals for comparison purposes. This would result in the prioritisation for conservation action of 373 DD or unassessed species, 271 nonthreatened (LC, NT) species, and 296 species that are threatened but with high uncertainty in their EDGE2 scores. The EDGE2 protocol enables us to instead delineate species of high importance based on their Red List category and levels of uncertainty and allocate species that fail to meet the criteria to the EDGE Watch List or EDGE Research List where applicable.

## Future directions

While our EDGE2 protocol synthesises numerous advances in the field of phylogenetically informed conservation prioritisation, it also provides a clear picture of what further research is now needed to improve the various components of the framework. There are, for example, numerous ways to incorporate extinction risk weightings into the protocol [44,45,56,72,78]. As our understanding of extinction risk improves, we certainly expect this component of EDGE2 to be revisited, with the ultimate goal of a process-based and biologically accurate calculation of globally threatened PD.

The incorporation of species lacking extinction risk data into global assessments of biodiversity loss is increasingly recognised as important [49,50,72,79–83]. Here, our approach to the incorporation of species lacking adequate extinction risk information was designed to retain simplicity and applicability to groups about whose extinction risk we still know relatively little (e.g., invertebrates, plants, and fungi). There are, however, other possible approaches to assigning GE2 scores to DD/NE species, for instance, drawing them in proportion to the observed frequency of extinction risk categories of either the assessed members of the clade or species that transitioned from DD to a data-sufficient category, or using extinction risk correlates to inform the imputed extinction risk of species [50]. Further, other available extinction risk information, such as estimates from predictive modelling or Population Viability Analyses, could provide valuable tools in parameterising uncertainty for species lacking Red List data, and the protocol is designed so such data could one day replace the coarser Red List categories if desirable. These options require further exploration in the context of EDGE2 and phylogenetic conservation more generally.

The capability of EDGE2 to generate comprehensive prioritisations for clades for which we lack comprehensive data—which, realistically, is all large clades of animals, plants, and fungi—provides the possibility for EDGE prioritisations to be developed for large regions of the Tree of Life, beyond those clades already assessed under the original EDGE approach (amphibians, birds, corals, reptiles, sharks and rays, gymnosperms). Continuing advances in open-sourced estimates of the Tree of Life [84] and their synthesis with extinction risk data [85], paired with the potential for increased IUCN Red Listing of large groups of species, means EDGE prioritisations for large portions of the Tree of Life are a realistic goal. However, work is still required to ensure maximum taxonomic agreement between all sources of data to ensure that the EDGE philosophy of maximising species inclusion is maintained.

Further, given that the vast majority of a species’ ED2 and EDGE2 scores are contributed by branches closest to the tips of the phylogenetic tree (S1 Fig), EDGE2 scores can be considered comparable across various clades when calculated from separate phylogenetic trees (e.g., lepidosaurs, crocodilians, and turtles), as the deep internal branches will contribute trivial amounts to ED2 scores due to the inclusion of PD complementarity [86]. This advance provides us with the option to generate and explore the use of EDGE Lists of species from disparate clades based on other thematic or taxonomic criteria (e.g., EDGE Mangroves or EDGE Pollinators).

## Conclusions

EDGE2 arrives at the inception of a major new framework for biodiversity conservation that the Parties to the CBD adopted in 2022. This vision includes a strident new call to turn the tide for species conservation, specifically to prevent further species extinctions, to stabilise net extinction risk, and to drive an increase in average population size. We believe the EDGE approach has a critical role to play in achieving this ambitious plan, especially in helping to prioritise investments in conservation efforts. However, the science behind the original EDGE metric has evolved considerably since the metric was originally proposed. By developing EDGE2, we hope to ensure that, as theory and data availability for conservation prioritisation continues to grow, more initiatives can implement conservation action underpinned by robust priority setting until another consensus view is eventually required. Our new EDGE2 protocol provides EDGE2 scores with associated measures of uncertainty, and membership of EDGE lists including new “watch” and “research” lists, for all species in a clade. One of the great strengths of EDGE is its positive interactions with the wider scientific community, and our workshop and ongoing collaboration have allowed us to include that community in the EDGE2 process. We feel that EDGE2 is an example of how academic and applied conservation biologists can work together to guide effective priority-setting that has the best chance of preserving the biodiversity upon which humanity depends.

## Supporting information

S1 Text Supporting information, detailed methods, and additional results for the main text section “Introducing the EDGE2 protocol.”. (DOCX)

S2 Text Supporting information, detailed methods, and additional results for the main text section “EDGE2 real-world application.”. (DOCX)

S1 Data EDGE2 scores for the world’s mammals, and the various lists produced under the EDGE2 protocol. (XLSX)

S2 Data The underlying data for Figs 1, 3, 4, 5, S1, S2, S3, S4, S5 and S6. (XLSX)

S1 Fig Distributions of ED2 and EDGE2 scores for mammals. The distribution of (**a**) the percentage of species’ ED2 scores contributed by their terminal branch lengths (TBL) alone; (**b**) ED2 scores; and (**c**) EDGE2 scores, for all mammals. Panels b and c are presented on a log-scale along the horizontal axis. The data underlying this Figure can be found in S2 Data.
(TIF)

S2 Fig The relationship between measures of ED. Relationship between median (**a**) TBL and ED2 scores; (**b**) ED1 and ED2 scores; and (**c**) TBL and ED1 scores, for all mammals. The data underlying this Figure can be found in S2 Data. ED, evolutionary distinctiveness; TBL, terminal branch length.
(TIF)

S3 Fig Relationship between EDGE1 and EDGE2 rankings. The EDGE2 ranks (x-axis) and EDGE1 ranks (y-axis) for (**a**) all mammals with data-sufficient and extant IUCN Red List assessments; and (**b**) all EDGE2 Species (above-median EDGE2 for 95% of iterations and threatened on IUCN Red List; see “EDGE2 Framework”). More details in S2 Text. The data underlying this Figure can be found in S2 Data.
(TIF)

S4 Fig Proportion of EDGE2 captured by increasing numbers of species. Cumulative percentage of total EDGE2 captured by increasing the number of species captured, from the mammal species with the highest EDGE2 score to the lowest. EDGE2 scores are cumulatively summed from the highest score to the lowest until all EDGE2 scores are captured. The data underlying this Figure can be found in S2 Data.
(TIF)

S5 Fig Changes in EDGE ranks between EDGE1 and EDGE2 for Artiodactyla. The EDGE1 and EDGE2 ranks of mammal species for which the ZSL’s EDGE of Existence programme has funded conservation projects, mapped onto the phylogenetic tree of artiodactylids, extracted from a tree at random from the 1,000-tree distribution used to calculate EDGE2 scores (see S2 Text). Colours of phylogenetic branches represent EDGE ranks, from highest priority to lowest priority, for terminal branches. For internal branches, colours represent the median rank of all descendant species. Colours around species images represent IUCN Red List status: purple = Extinct in the fWild; red = CR; orange = EN; yellow = VU. Downward arrows represent species that are lower priority under EDGE2 compared with EDGE1; upward arrows represent species that are higher priority under EDGE2. The data underlying this Figure can be found in S2 Data. *Image credits*: *Elaphurus davidianus* and *Hippopotamus amphibius*: ZSL; *Nanger dama*: Tim Wacher; *Catagonus wagneri*: Proyecto Quimilero. CR, Critically Endangered; EDGE, Evolutionarily Distinct and Globally Endangered; EN, Endangered; IUCN, International Union for Conservation of Nature; VU, Vulnerable; ZSL, Zoological Society of London.
(TIF)

S6 Fig EDGE1 and EDGE2 ranks of EDGE programme mammal projects. The EDGE1 and EDGE2 ranks of mammal species for which the ZSL’s EDGE of Existence programme has supported conservation projects. The data underlying this Figure can be found in S2 Data. EDGE, Evolutionarily Distinct and Globally Endangered; ZSL, Zoological Society of London.
(TIF)

## Abbreviations

CBD: Convention on Biological Diversity
CR: Critically Endangered
DD: Data Deficient
ED: evolutionary distinctiveness
EDGE: Evolutionarily Distinct and Globally Endangered
EN: Endangered
GBF: Global Biodiversity Framework
GE: global endangerment
HED: heightened evolutionary distinctiveness
HEDGE: Heightened EDGE
IPBES: Intergovernmental Science-Policy Platform for Biodiversity and Ecosystem Services
IUCN: International Union for Conservation of Nature
LC: Least Concern
NE: Not Evaluated
NT: Near Threatened
PD: phylogenetic diversity
SSC: Species Survival Commission
TBL: terminal branch length
VU: Vulnerable
ZSL: Zoological Society of London
