# Supplementary material for: The EDGE2 protocol: Advancing the prioritisation of Evolutionarily Distinct and Globally Endangered species for practical conservation action
Source: PLoS Biol. 2023 Feb 28;21(2):e3001991. doi: 10.1371/journal.pbio.3001991 (PMC9974121; doi:10.1371/journal.pbio.3001991)
Supplement: S1 Text — (DOCX) [file pbio.3001991.s001.docx]

S1 Text.

Supporting information, detailed methods, and additional results for the main text section ‘Introducing the EDGE2 protocol’.

## PD complementarity

PD complementarity is the explicit consideration of the interplay of extinction risk of related species on the expected future contributions to future PD of a shared phylogenetic branch [1]. For example, consider a species that is Critically Endangered, but where the sister species (i.e. they share the same most recent internal phylogenetic branch; e.g. species A and B in Fig A, below) is Least Concern. Under the original ED formulation, the contribution of the shared ancestral branch to the ED of the Critically Endangered species is unaffected by the extinction risk of the sister species. However, when we incorporate PD complementarity, the contribution of the shared internal branch to the ED score of the Critically Endangered species is affected by the extinction risk of the sister species. As the sister species is Least Concern, and unlikely to become extinct, the shared internal branch is quite safe, and thus contributes less to the ED score than if the sister species was also threatened with extinction. Had the sister species been Critically Endangered, the internal branch would have been at much greater risk and thus contribute more to the ED score of our focal species. Thus, in our idealised scenario, ED2 measures the expected phylogenetic contribution of a species based on the probability that it will become the sole extant descendant of any of its ancestral phylogenetic branches (i.e. the probability that all other descendants become extinct) at a future point in time (see Fig A for an illustrated example).


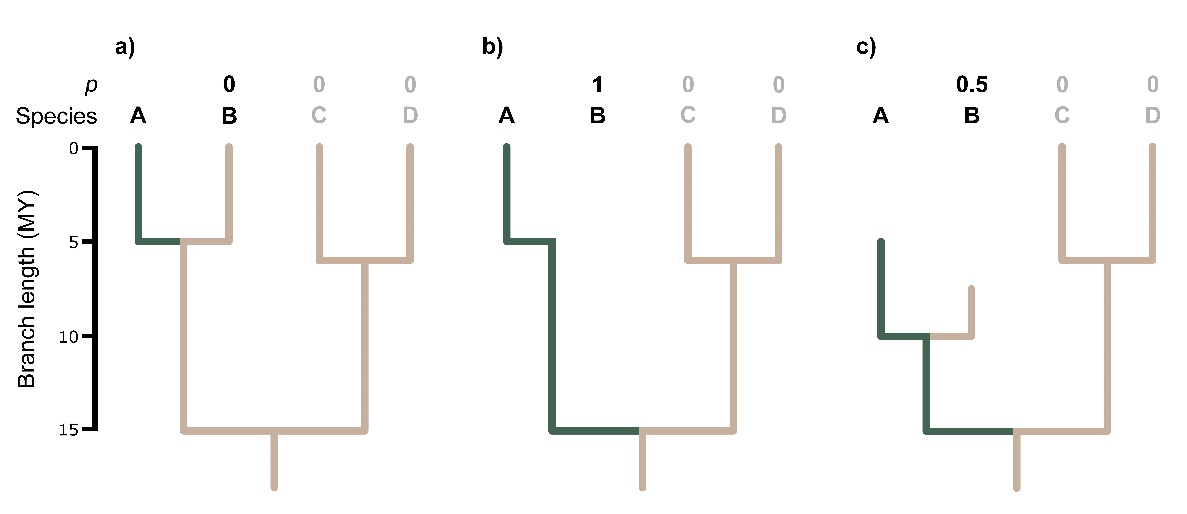


***Fig A. Incorporating PD complementarity into ED2 calculations under different extinction scenarios.*** *When the extinction risk (p; defined in the ED2 equation in the main text) of all species is 0, as in panel (a), the ED2 of species A is limited to the length of its terminal branch (blue branch), as there is zero probability of species B becoming extinct, and therefore zero probability their shared internal branch will contribute to species A's terminal branch length in the future. However, if species B becomes extinct (p = 1 in panel b), its terminal branch is lost and species A becomes the sole representative of their shared internal branch, which then becomes an extension of species A’s terminal branch (blue branches in b). When the extinction risk of species B is between 0 and 1, as when p = 0.5 in panel (c), this represents a 50% probability that species B becomes extinct before a certain point in the future. There is therefore a 50% chance that species A, when its extinction risk is not considered, will become the sole representative of the shared internal branch between species A and B, and thus the ED2 of species A is now its terminal branch length (solid blue branch, panel c) plus 50% of the shared branch’s length (dashed blue and green branch, panel c).*

## Terminal versus internal branches

One consequence of ED2’s explicit incorporation of PD complementarity is the interaction between phylogenetic structure and extinction risk, through the multiplication of branch lengths and probabilities of extinction. This interaction naturally down-weights the contribution of deeper phylogenetic branches to the ED2 scores of species; the more descendant species there are, the smaller the probability that a branch will be lost. This is not necessarily a concern: most species-based PD metrics are heavily weighted by the branches closest to the tips [2,3], and terminal branches have long been recognised as a valuable measure of distinctiveness [1,4,5]. However, a key area of consensus at the workshop was that the contribution of internal branches to biodiversity should be valued in any EDGE protocol.

To highlight the relative contributions of terminal and internal branches to ED2 scores, we explicitly decomposed ED2 into two components: (1) the terminal branch length, which represents the current PD exclusive to that species, and therefore its current distinctiveness [1] (Fig A); and (2) the cumulative contribution of all internal branches, accounting for PD complementarity, which represents the additional PD for which the species is expected to be responsible into the future given the current extinction risk of its relatives (Fig A). This facilitates the identification of species that are expected to be responsible for a large amount of PD from internal branches, indicating that they are part of highly distinctive clades with widespread elevated extinction risk. The decomposition of ED2 scores also permits terminal branch lengths—which are highly correlated with original ED [3]—to be used as a minimum estimate of ED2 when deeper phylogenetic structure is highly uncertain.

## Values of extinction risk

We chose to map GE2 to IUCN Red List extinction risk categories in a way that preserves the same intervals between Red List categories as the original GE index, where each step-wise increase in Red List category results in a doubling of extinction risk [6]. This followed the workshop principle of retaining components from the original EDGE approach unless we have a compelling reason otherwise. It also means that we only needed to establish a probability of extinction for Critically Endangered species and the rest would follow.

At our workshop, we found no compelling argument as to what time horizon would be most appropriate for calculating probabilities of extinction (e.g. those of Mooers et al. [7]). Delegates could only agree to dismiss time horizons that were either too soon (e.g. 10 years) or too distant (e.g. 500 years) to be relevant for stimulating action. There are naturally subjective elements to any choice; it is tempting to advocate for time horizons farther into the future based on an intuition that conservation efforts are for long-term benefits and should therefore be protected from potential short-sighted decision-making. However, such arguments may not hold up to further scrutiny. Extinction risk information for species can be expected to be updated at the scale of years rather than decades; IUCN Red List assessments of extinction risk are deemed out-dated after 10 years [8]. Although timely reassessments of species can be difficult to achieve [9,10], we still see this as reasonable justification for choosing a time horizon on the order of decades rather than centuries.

We therefore opted to tie the doubling feature of GE’s extinction risk to the 50-year time horizon specified in Mooers et al. [7] (a study notable for examining the impact of time-horizon and *p* definition on conservation prioritisation), with Critically Endangered (CR) mapped to an extinction risk weighting of 0.97. Endangered (EN) species therefore receive a weighting of 0.485, Vulnerable (VU) = 0.2425, Near Threatened (NT) = 0.12125, and Least Concern (LC) = 0.060625.

Our choice of an absolute extinction risk value for CR species tied to the 50-year time horizon, combined with the principle of doubling extinction risk with each increase in Red List category from the original EDGE metric, provides a set of values to capture the original properties of the GE component. That is, the extinction risk values are of high absolute value and risk is halved with every down listing, consistent with the original EDGE formulation [6,11]. At the workshop we considered many other alternatives (e.g.[7]), but no single approach received enough support to justify a further departure from the original EDGE approach. Thus, the concept of GE2 represents a clear conceptual advance in some respects, allowing for the incorporation of probabilities of extinction, while retaining the behaviour of original GE scores in other respects where current knowledge is not enough to make an unequivocal improvement.

## Incorporating GE2 uncertainty

We developed a new approach to incorporate uncertainty in GE2 and enable the inclusion of Data Deficient (DD) and Not Evaluated (NE) species, the inclusion of which has been demonstrated to reduce bias in estimates of phylogenetically-informed prioritisations [12]. Our approach is based on the idea that if we were to rank species by their true probability of extinction, the probabilities will change smoothly reflecting biological processes, and not jump as we move between discrete, arbitrary, human-inferred Red List categories (Fig 1 in main text).

We can therefore reconstruct a smooth curve based on realistic constraints: (1) *p* bounded by a maximum of 0.9999 (almost certain to go extinct) at one extreme and 0.0001 (safe) at the other extreme; (2) *p* must be increasing on account of being ranked by increasing extinction risk; and (3) the median *p* drawn from the portion of distribution corresponding to a Red List category must equal the chosen *p* for that category - the median of all possible *p* draws for a CR species. should remain equal to 0.97 after the process (Fig 1 in main text). The portion of the distribution assigned to each category is equal in size, though this could also vary in size in proportion to the observed distribution of Red List categories for a taxonomic group. We elected to use equal bands for each category to allow the generated distribution to be more widely applicable and consistent across multiple taxa, for which the distribution of Red List categories differs. For each species, *p* is drawn for each species from the rank plot by selecting at random a value on the x-axis that falls within the range for the corresponding Red List category (Fig 1 in main text).

For unassessed NE and DD species, *p* is drawn from the entire distribution completely at random under the assumption that, although we do not know where NE and DD species will be located along the curve, it must be at some point between 0 and 1, as with other extant species. Thus we generate highly uncertain scores for NE and DD species that have a median value equivalent to elevated extinction risk (VU) when taken over a large number of iterations [13,14]. For species listed as Possibly Extinct (CR(PE)), Possibly Extinct in the Wild (CR(PEW)) and Extinct in the Wild (EW), *p* is drawn from the corresponding CR values, and all Extinct (EX) species have *p* = 1 by definition. The treatment of PE, PEW, and EW as distinct from EX, with *p* equal to CR, deviates from the Red List Index approach [15]; we took this approach to reflect the fact that PE/PEW/EW species, unlike EX species, have the potential for recovery in the wild.

We draw *p* for all species again in each iteration of calculating EDGE2 scores and can therefore capture uncertainty in the final results of ED2 and GE2 (= *p*) while retaining the condition that the median GE2 score for each species aligns with the score corresponding to its Red List category. Other sources of uncertainty, for example in the Red List categorisation for the species themselves, and in the treatment of CR(PE), CR(PEW), and EW species, are subjects for future research. For clades where only a single well-supported consensus phylogenetic tree containing all recognised species is available, ED2, and thus *p*, must still be calculated a large number of times to adequately capture the uncertainty in *p* (100 < n ≤ 1000 to mirror approaches to capture phylogenetic uncertainty [16–18]) .

# References

1. Faith DP, Reid CAM, Hunter J. Integrating Phylogenetic Diversity, Complementarity, and Endemism for Conservation Assessment. Conserv Biol. 2004;18: 255–261. doi:10.1111/j.1523-1739.2004.00330.x

2. Redding DW, Hartmann K, Mimoto A, Bokal D, DeVos M, Mooers A. Evolutionarily distinctive species often capture more phylogenetic diversity than expected. J Theor Biol. 2008;251: 606–615. doi:10.1016/j.jtbi.2007.12.006

3. Redding DW, Mazel F, Mooers A. Measuring evolutionary isolation for conservation. PLoS One. 2014;9: e113490. doi:10.1371/journal.pone.0113490

4. Faith DP. Conservation evaluation and phylogenetic diversity. Biol Conserv. 1992;61: 1–10. doi:10.1016/0006-3207(92)91201-3

5. Tucker CM, Aze T, Cadotte MW, Cantalapiedra JL, Chisholm C, Díaz S, et al. Assessing the utility of conserving evolutionary history. Biol Rev. 2019;94: 1740–1760. doi:10.1111/brv.12526

6. Isaac NJB, Turvey ST, Collen B, Waterman C, Baillie JEM. Mammals on the EDGE: Conservation priorities based on threat and phylogeny. PLoS One. 2007;2: e296. doi:10.1371/journal.pone.0000296

7. Mooers A, Faith DP, Maddison WP. Converting endangered species categories to probabilities of extinction for phylogenetic conservation prioritization. PLoS One. 2008;3: 1–5. doi:10.1371/journal.pone.0003700

8. IUCN. IUCN Red List Categories and Criteria version 3.1. Cambridge, United Kingdom: IUCN; 2001.

9. Rondinini C, Di Marco M, Visconti P, Butchart SHM, Boitani L. Update or Outdate: Long-Term Viability of the IUCN Red List. Conserv Lett. 2014;7: 126–130. doi:10.1111/conl.12040

10. Tapley B, Michaels CJ, Gumbs R, Böhm M, Luedtke J, Pearce-Kelly P, et al. The disparity between species description and conservation assessment: A case study in taxa with high rates of species discovery. Biol Conserv. 2018;220: 209–214. doi:https://doi.org/10.1016/j.biocon.2018.01.022

11. Isaac NJB, Redding DW, Meredith HM, Safi K. Phylogenetically-Informed Priorities for Amphibian Conservation. PLoS One. 2012;7: 1–8. doi:10.1371/journal.pone.0043912

12. Veron S, Penone C, Clergeau P, Costa GC, Oliveira BF, Sao-Pedro VA, et al. Integrating data-deficient species in analyses of evolutionary history loss. Ecol Evol. 2016;6: 8502–8514. doi:https://doi.org/10.1002/ece3.2390

13. Bland LM, Collen B, Orme CDL, Bielby J. Predicting the conservation status of data-deficient species. Conserv Biol. 2015;29: 250–259. doi:https://doi.org/10.1111/cobi.12372

14. González-del-Pliego P, Freckleton RP, Edwards DP, Koo MS, Scheffers BR, Pyron RA, et al. Phylogenetic and Trait-Based Prediction of Extinction Risk for Data-Deficient Amphibians. Curr Biol. 2019;29: 1557-1563.e3. doi:https://doi.org/10.1016/j.cub.2019.04.005

15. Butchart SHM, Stattersfield AJ, Bennun LA, Shutes SM, Akçakaya HR, Baillie JEM, et al. Measuring Global Trends in the Status of Biodiversity: Red List Indices for Birds. PLOS Biol. 2004;2: e383. doi:10.1371/journal.pbio.0020383

16. Collen B, Turvey ST, Waterman C, Meredith HMR, Kuhn TS, Baillie JEM, et al. Investing in evolutionary history: implementing a phylogenetic approach for mammal conservation. Philos Trans R Soc Lond B Biol Sci. 2011;366: 2611–2622. doi:10.1098/rstb.2011.0109

17. Jetz W, Thomas GH, Joy JB, Redding DW, Hartmann K, Mooers AO. Global Distribution and Conservation of Evolutionary Distinctness in Birds. Curr Biol. 2014;24: 919–930. doi:10.1016/j.cub.2014.03.011

18. Weedop KB, Mooers AØ, Tucker CM, Pearse WD. The effect of phylogenetic uncertainty and imputation on EDGE Scores. Anim Conserv. 2019;22: 527–536. doi:10.1111/acv.12495
