# Supplementary material for: The EDGE2 protocol: Advancing the prioritisation of Evolutionarily Distinct and Globally Endangered species for practical conservation action
Source: PLoS Biol. 2023 Feb 28;21(2):e3001991. doi: 10.1371/journal.pbio.3001991 (PMC9974121; doi:10.1371/journal.pbio.3001991)
Supplement: S2 Text — (DOCX) [file pbio.3001991.s002.docx]

S2 Text.

Supporting information, detailed methods, and additional results for the main text section ‘EDGE2 real world application’.

## Example for the world’s mammals

We applied the principles of the EDGE2 framework to develop a revised global EDGE prioritisation for the world’s mammals. We selected a random sample of 1,000 phylogenetic trees from the recently-published [1] 10,000-tree ‘pseudo-posterior’ distribution of birth-death node-dated trees comprising 5,911 extant and extinct mammal species. Our taxonomy for mammals was taken from version 1.1 of The Mammal Diversity Database [2], from which we determined 6,253 extant valid mammal species as of 01/05/2020. We matched this taxonomy with both that of our distribution of phylogenetic trees and with IUCN Red List data for 5,853 mammal species (as of 11/05/2020)[3]. We removed all Extinct species from each tree to remove their influence on the ED and EDGE scores of closely-related taxa [4] and to more accurately estimate expected PD loss for extant species only, though for EDGE2 prioritisations their influence can also be removed by setting their extinction risk weighting to 1. To ensure EDGE2 priority setting for mammals was comprehensive, we then inserted all valid mammal species—according to our taxonomy—missing from the phylogenetic trees (421 spp.; 6.7% of mammals) to their respective genus (or family for species with no congeners in the tree) using the ‘*congeneric.impute*’ function in the R package ‘*pez*’ [5]. This approach approximates a previous method [6] for imputing missing species into a phylogeny. We generated a distribution of 1,000 trees comprising all extant mammal species at the time of analysis.

To generate a distribution of probabilities of extinction for each IUCN Red List category we fitted a quartic curve through the five median values for each Red List category (LC = 0.060625, NT = 0.12125, VU = 0.2425, EN = 0.485, CR + EW = 0.97) and bounded the resulting curve to return values between 0.0001 and 0.9999. We calculated ED2 and EDGE2 scores across our distribution of 1,000 trees and for each tree we selected new extinction risk weightings for each species at random from the distribution of GE2 scores tied to the species’ Red List category. This resulted in 1,000 ED2 and EDGE2 scores for all species. For currently NE and DD species we drew their GE2 scores from the entire distribution of GE2 values, which had a median of 0.232, comparable to that of the VU category (Fig A), and consistent with trait and range size-based predictions that indicate elevated extinction risk amongst DD species [7,8]. We then generated the EDGE Species List, as well as the Watch, Research and Borderline EDGE Lists for mammals (following Fig 2 in main text). All ED2 and EDGE2 scores are given to the nearest million years to reflect the degree of accuracy appropriate for these data given the uncertainty in phylogenetic and extinction risk estimates. Functions to generate GE2 and EDGE2 scores are available online to speed up the generation of future EDGE2 lists (<https://github.com/rgumbs/EDGE2/>).


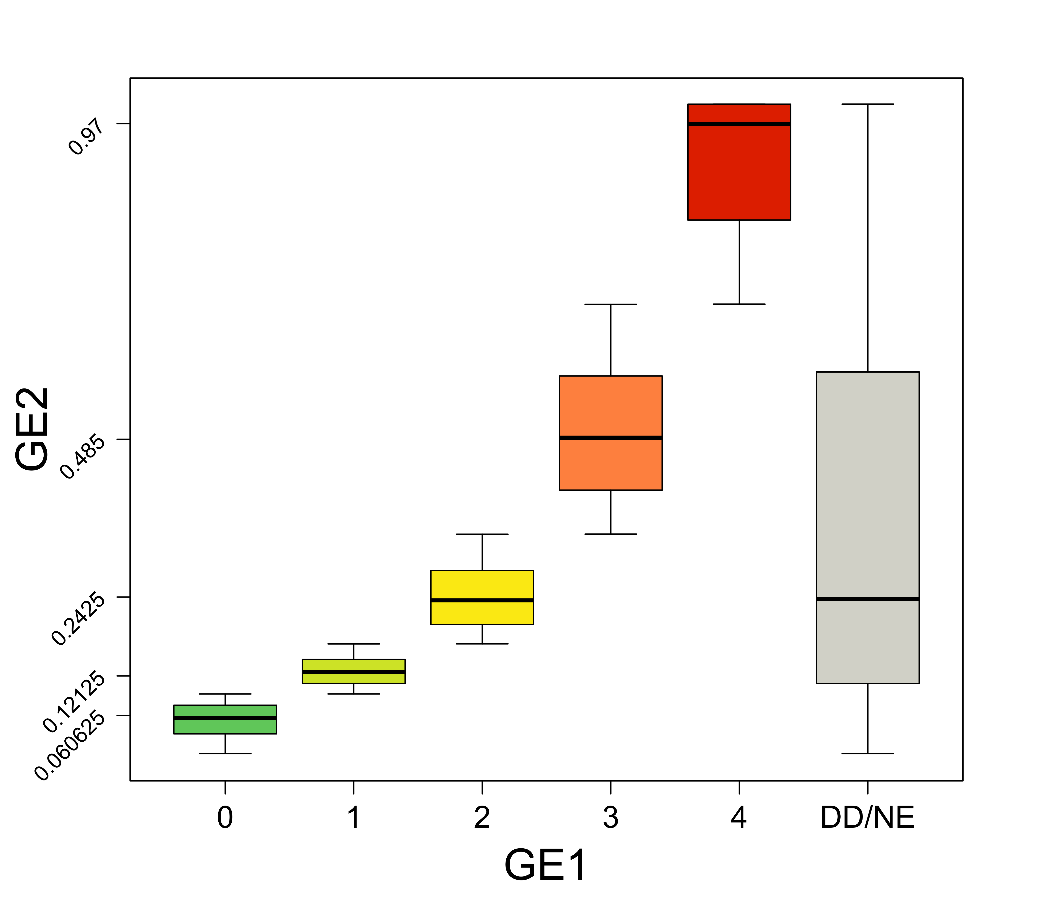


***Fig A. Mapping GE2 to extinction risk weightings.*** *Distribution of GE2 scores from which extinction risk weightings were selected for each Red List category, corresponding to their original GE1 scores in EDGE1. For GE1 scores, 0 = LC, 1 = NT, 2 = VU, 3 = EN, 4 = CR. DD and NE species were not given a GE1 score in the original EDGE1 metric.*

## Imputation analyses

One concern of using imputed phylogenetic trees, as we have here, is the impact of the imputed species on the accuracy of the ED and EDGE scores of the species for which we have molecular data [9]. To explore this, we calculated EDGE2 rankings for all species in both the molecular-data-only phylogenetic tree of Upham et al. [1] (hereafter ‘molecular-only tree’) and the larger tree containing species they imputed using taxonomy (hereafter ‘taxonomy-imputed tree’). We correlated these rankings with one another for all species present in both trees, and also correlated both sets of rankings from the Upham et al. trees with those from our phylogenetic trees with the remaining 421 missing species imputed (hereafter ‘fully-imputed tree’). To shed light on the impacts of our imputation on conservation priorities, we determined how many species in the top 100 EDGE2 ranks have: i) molecular data; ii) were imputed by Upham et al. [1]; and iii) were imputed in our study. Finally, to determine whether the imputation of species increased variation in ED2 and EDGE2 scores, we calculated the variance in ED2 and EDGE2 scores for all species across the 1,000 trees and correlated these against the proportion of each genus that was included in the phylogenetic tree using genetic data.

The median EDGE2 rankings from our fully-imputed mammal phylogenetic trees are strongly correlated with those from the taxonomy-imputed tree (ρ = 0.99, df = 5401, p < 0.0001; Fig B). The EDGE2 rankings from our fully-imputed trees are strongly correlated with those from the molecular-only tree (ρ = 0.79, df = 4020, p < 0.0001), and the sets of EDGE2 rankings from the taxonomy-imputed and molecular-only trees are similarly correlated (ρ = 0.80, df = 4020, p < 0.0001; Fig B). Eighty-eight of the 100 highest ranking EDGE2 mammals were placed in the phylogenetic tree using molecular data, and the remaining 12 species were imputed based on taxonomy by Upham et al. [1]. Just six of the 633 EDGE2 Species (above median EDGE2 for 95% of iterations and VU, EN, CR) were imputed in this study.


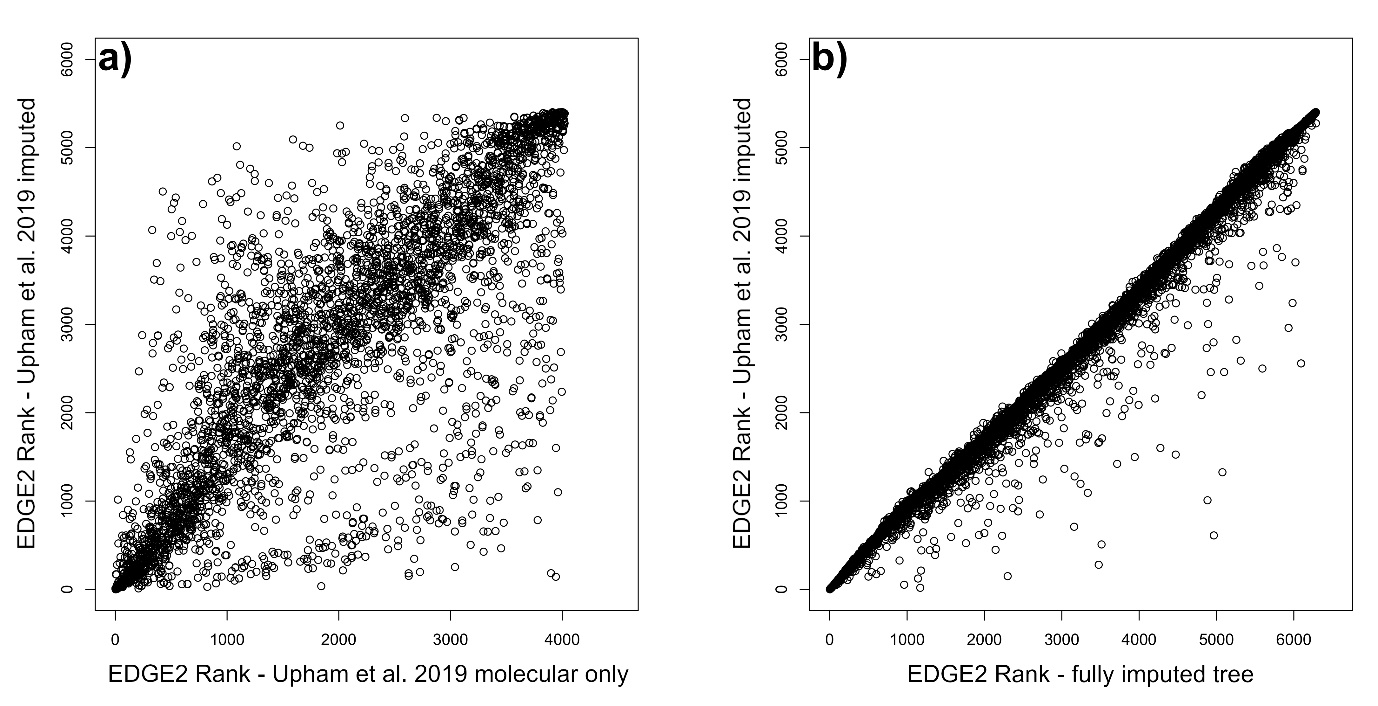


***Fig B.*** *Relationship between the median EDGE2 ranks from (a) the taxonomy-imputed tree and molecular-only tree of Upham et al. (2019); and (b) the taxonomy-imputed tree of Upham et al.* [1] *(y-axis) and the fully-imputed tree generated from this study (x-axis).*

Species imputed here have variance in both ED2 and EDGE2 scores comparable to those imputed by Upham et al. [1], though both exhibit greater variance than species placed using molecular data (Fig C). Variance decreases as molecular coverage of a genus increases for both ED2 (ρ = -0.394, df = 6251, p < 0.0001) and EDGE2 scores (ρ = -0.221, df = 6251, p < 0.0001). This increased variance meant that of 421 species imputed here with EDGE2 scores above the median, just 7 (1.6%) were above median EDGE2 in 95% or more iterations, compared with 33.8% of species with molecular data. This increased variance limits the potential for species for which we lack adequate understanding of their evolutionary distinctiveness to dominate EDGE2 priority lists unless the species are part of particularly ancient and species-poor clades. The inclusion of otherwise missing species also serves to reduce the ED2 and EDGE2 scores in clades where a small proportion of described species are included, such as the genus *Dromiciops*, where two recently-described species were absent from the Upham et al. [1] phylogenetic tree [10], thus reducing the potential for species to be incorrectly identified as priority species due to overestimation of their evolutionary distinctiveness.


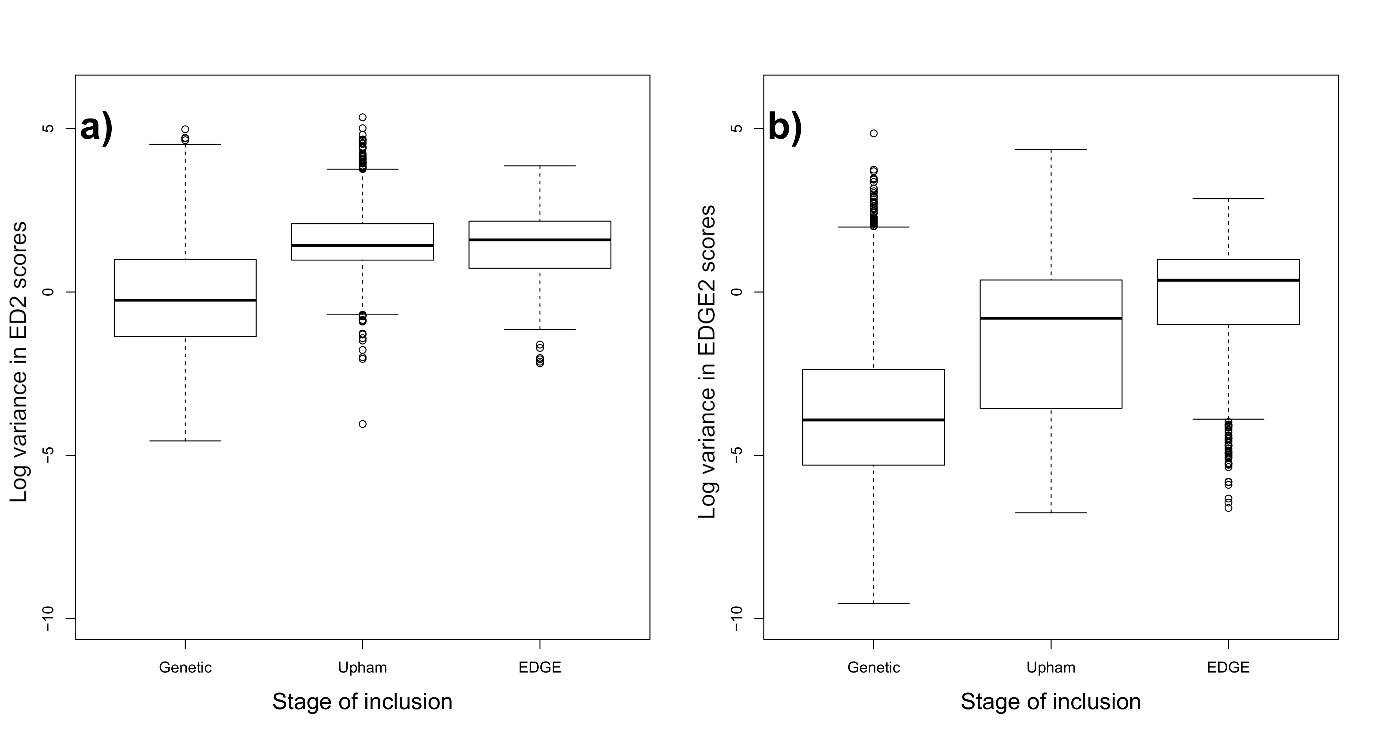


***Fig C:*** *Variance in the (a) ED2 and (b) EDGE2 scores for species inserted into the phylogenetic tree using either genetic data, taxonomy by Upham et al.* [1]*, or taxonomy here, calculated across 1,000 phylogenetic trees.*

## Comparing EDGE priorities

For comparison with the new EDGE2 protocol, we calculated original EDGE (EDGE1) scores for all species with data-sufficient Red List categories (LC, NT, VU, EN, CR), and limited the EDGE2 comparison dataset to species assigned to the same Red List categories. We correlated EDGE2 ranks with EDGE1 ranks and calculated the percentage overlap between the highest 100 ranking species of the two. Finally, we explored how the magnitude of change in EDGE rank from EDGE1 to EDGE2 was related to the ranking priority of the species, by correlating the difference between EDGE1 and EDGE2 ranks with the absolute EDGE2 rank, to determine whether highly ranked EDGE2 species varied less in their rank change than lower-ranked (lesser priority) EDGE2 species.

Sixty-seven of the 100 highest-ranking threatened species under EDGE2 are also in the top 100 ranking species under EDGE1. A further 30 species from the top 100 EDGE2 ranks are outside the top 100 EDGE1 ranks, but still qualify as priority EDGE species under the original criteria. The three species not considered as EDGE species under the original criteria are *Habromys lepturus*, *H. ixtlani*, and *Simias concolor*; they have ED1 scores below the median but EDGE2 scores above median. All of the top 100 EDGE species identified using the EDGE1 framework are captured in the 645 EDGE2 Species, and 97 of the top 100 EDGE2 species are captured in the 529 EDGE1 Species.

## Comparing measures of ED

The proportion of the ED2 score contributed by the TBL indicates the relative importance of the current distinctiveness of the species in question (large proportion of ED2 contributed by TBL would be a species with high ‘PD endemism’, *sensu* [11]) versus the heightened potential future responsibility of the species for internal branches due to the high probability that the other species descending internal branches will become extinct (large proportion of ED2 contributed by internal branches). We therefore calculated the terminal branch length (TBL) for each species in each tree and ran a correlation of ED2 and TBL to explore whether larger ED2 scores were associated with longer terminal branches or with the component corresponding to interior branches. To determine whether species with longer terminal branches receive a greater proportion of their ED2 from their TBL than those with shorter branches, we correlated TBL and the proportion of ED2 contributed by the TBL for each species.

We calculated original ED (hereafter ED1) scores across the 1,000 trees for all species and correlated the median ED1 scores for each species with their ED2 scores. We correlated ED2 ranks with ED1 ranks and calculated the percentage overlap between the highest 100 ranking species between the two. We also correlated the change in rank from ED1 to ED2 for each species with the proportion of ED2 contributed by TBL for each species to determine whether species with greater contributions from their terminal branches rank more highly under ED2 than ED1, given the latter accounts for the complementary contribution of close relatives to the persistence of internal branches.

ED2 is strongly positively correlated with terminal branch length (TBL; Pearson’s product-moment: r = 0.95, df = 6251, p < 0.0001; S2 Fig). ED2 is strongly positively correlated with original ED values calculated following Isaac et al. [12] (hereafter ED1; r = 0.829, df = 6251, p < 0.0001; S2 Fig), though ED2 scores are significantly smaller than those of ED1 (Wilcoxon signed rank: V = 5.58 x10^4^, df = 6251, p < 0.0001). This is due to the greater contribution of internal branches to ED1 scores and the fact that ED1 scores sum to PD whilst ED2 scores sum to a smaller figure (approximating total expected PD loss for the clade).

The median proportion of ED2 contributed by the TBL of the species is 87.2% (range: 4.8% - 99.9%; S1 Fig), and this proportion is moderately positively correlated with TBL alone, with high significance also driven by the large sample size (r = 0.326, df = 6251, p < 0.0001). The proportion of ED2 contributed by TBL is strongly negatively correlated with the proportional change in ED1 and ED2 ranks (r = -0.886, df = 6251, p < 0.0001). Sixty-three of the 100-highest ranking species under ED2 are also in the 100 highest ranks for ED1. Change in ranking from ED1 to ED2 is moderately positively correlated with the proportion of ED2 contributed by TBL (Spearman’s rank: ρ = 0.296, df = 6251, p < 0.0001), again reflecting the greater weighting towards terminal branches under ED2.

## Transitioning from EDGE1 to EDGE2

We extracted the median terminal and internal branch contributions for all species during the calculation of EDGE2. For Fig 4 in the main text we extracted a subtree for each species from the 1,000 phylogenetic trees generated for the EDGE2 scores calculations that reflected the median terminal branch lengths for useful visualisation purposes. We used the EDGE1 ranks calculated for comparison with EDGE2 ranks to calculate the change in ranks from EDGE1 to EDGE2 for all threatened species featured in Fig 4 in the main text.

For mapping the global change in numbers of EDGE species at the national level we calculated the number of EDGE1 species present in each country, and the number of EDGE2 species present in each country, then calculated the difference between the two values. To determine whether an EDGE1 or EDGE2 species was present in a country, we used the ‘rredlist’ package in R [3] to extract the native and extant country occurrences listed for each species. Species featured in Fig 5 were identified as being only EDGE1 species (i.e. no longer EDGE species) or only EDGE2 species (new EDGE species), the full list of species is available in S1 Data and S2 Data.

To compare the EDGE1 and EDGE2 ranks of mammal species targeted by ZSL’s EDGE of Existence programme for conservation projects, we took the current ranks calculated here for EDGE1 and EDGE2, rather than the rank at the time of the commencement of the project. The species targeted by projects supported by the EDGE of Existence programme are listed on the programme’s website [13]. We calculated the median current EDGE1 and EDGE2 ranks for each species and whether it was an EDGE2 species today. We used a paired t-test of log-transformed rank data to determine whether there was a difference in the current EDGE1 and EDGE2 ranks of EDGE of Existence-supported species.

1. Upham NS, Esselstyn JA, Jetz W. Inferring the mammal tree: Species-level sets of phylogenies for questions in ecology, evolution, and conservation. PLOS Biol. 2019;17: e3000494. doi:10.1371/journal.pbio.3000494

2. Mammal Diversity Database. Mammal Diversity Database (Version 1.1) [Data set]. Zenodo; 2020. doi:10.5281/zenodo.4139788

3. IUCN. IUCN Red List of Threatened Species. Version 2020-1. 2020. Available: www.iucnredlist.org

4. Gumbs R, Gray CL, Wearn OR, Owen NR. Tetrapods on the EDGE: Overcoming data limitations to identify phylogenetic conservation priorities. PLoS One. 2018;13: e0194680. doi:10.1371/journal.pone.0194680

5. Pearse WD, Cadotte MW, Cavender-Bares J, Ives AR, Tucker CM, Walker SC, et al. pez: phylogenetics for the environmental sciences. Bioinformatics. 2015;31: 2888–2890. doi:10.1093/bioinformatics/btv277

6. Kuhn TS, Mooers A, Thomas GH. A simple polytomy resolver for dated phylogenies. Methods Ecol Evol. 2011;2: 427–436. doi:10.1111/j.2041-210X.2011.00103.x

7. Bland LM, Collen B, Orme CDL, Bielby J. Predicting the conservation status of data-deficient species. Conserv Biol. 2015;29: 250–259. doi:https://doi.org/10.1111/cobi.12372

8. González-del-Pliego P, Freckleton RP, Edwards DP, Koo MS, Scheffers BR, Pyron RA, et al. Phylogenetic and Trait-Based Prediction of Extinction Risk for Data-Deficient Amphibians. Curr Biol. 2019;29: 1557-1563.e3. doi:https://doi.org/10.1016/j.cub.2019.04.005

9. Weedop KB, Mooers AØ, Tucker CM, Pearse WD. The effect of phylogenetic uncertainty and imputation on EDGE Scores. Anim Conserv. 2019;22: 527–536. doi:10.1111/acv.12495

10. Burgin CJ, Colella JP, Kahn PL, Upham NS. How many species of mammals are there? J Mammal. 2018;99: 1–14. doi:10.1093/jmammal/gyx147

11. Faith DP, Reid CAM, Hunter J. Integrating Phylogenetic Diversity, Complementarity, and Endemism for Conservation Assessment. Conserv Biol. 2004;18: 255–261. doi:10.1111/j.1523-1739.2004.00330.x

12. Isaac NJB, Turvey ST, Collen B, Waterman C, Baillie JEM. Mammals on the EDGE: Conservation priorities based on threat and phylogeny. PLoS One. 2007;2: e296. doi:10.1371/journal.pone.0000296

13. ZSL EDGE of Existence. EDGE of Existence. 2022. Available: http://edgeofexistence.org/
