# Supplementary figures and images for: The EDGE2 protocol: Advancing the prioritisation of Evolutionarily Distinct and Globally Endangered species for practical conservation action
Source: PLoS Biol. 2023 Feb 28;21(2):e3001991. doi: 10.1371/journal.pbio.3001991 (PMC9974121; doi:10.1371/journal.pbio.3001991)

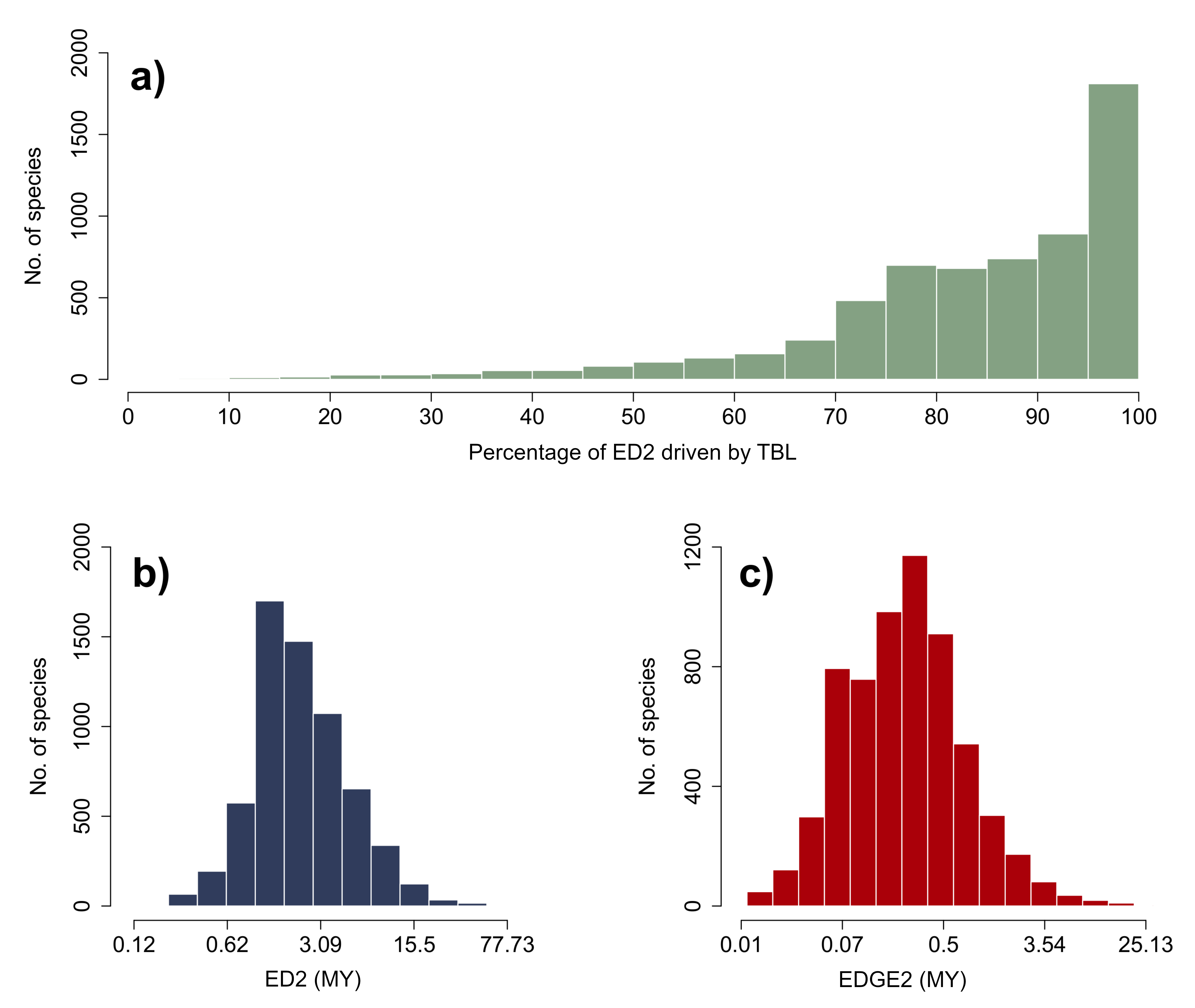

Supplement: S1 Fig — The distribution of (a) the percentage of species’ ED2 scores contributed by their terminal branch lengths (TBL) alone; (b) ED2 scores; and (c) EDGE2 scores, for all mammals. Panels b and c are presented on a log-scale along the horizontal axis. The data underlying this Figure can be found in S2 Data. (TIF) [file pbio.3001991.s005.tif]

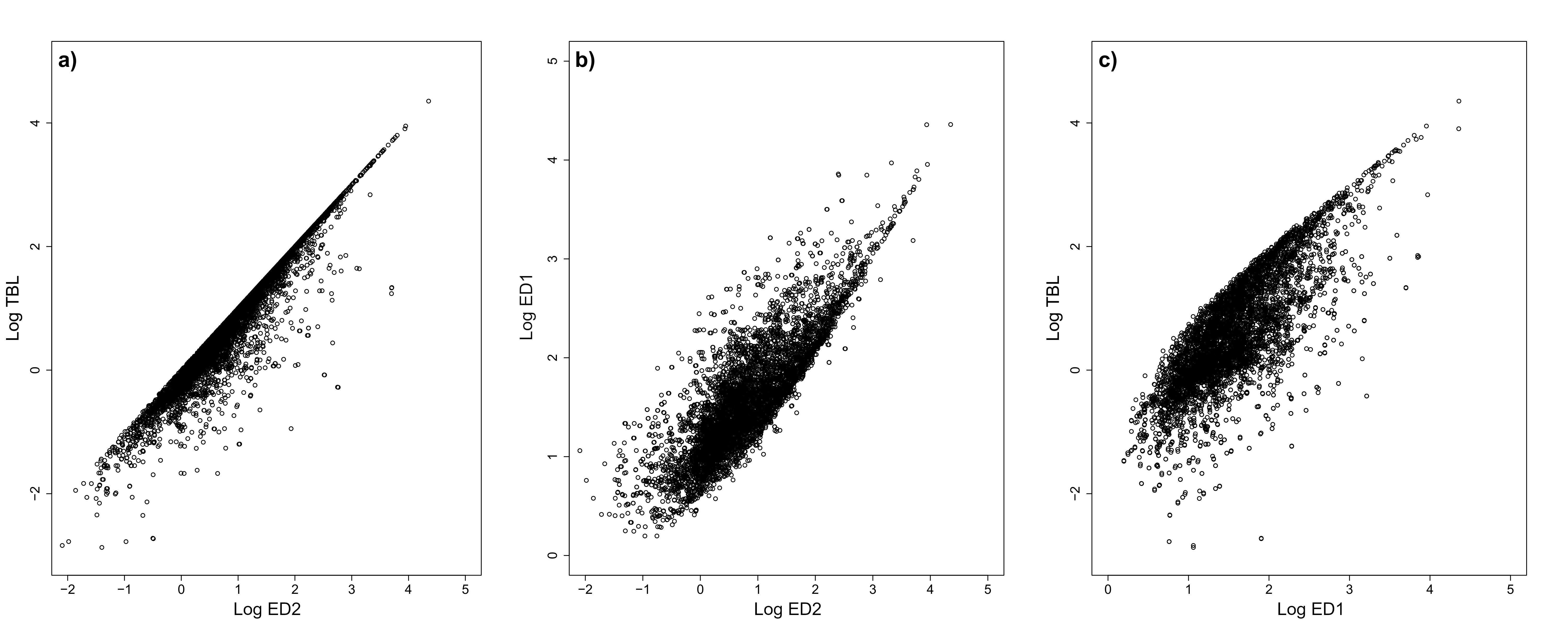

Supplement: S2 Fig — Relationship between median (a) TBL and ED2 scores; (b) ED1 and ED2 scores; and (c) TBL and ED1 scores, for all mammals. The data underlying this Figure can be found in S2 Data. ED, evolutionary distinctiveness; TBL, terminal branch length. (TIF) [file pbio.3001991.s006.tif]

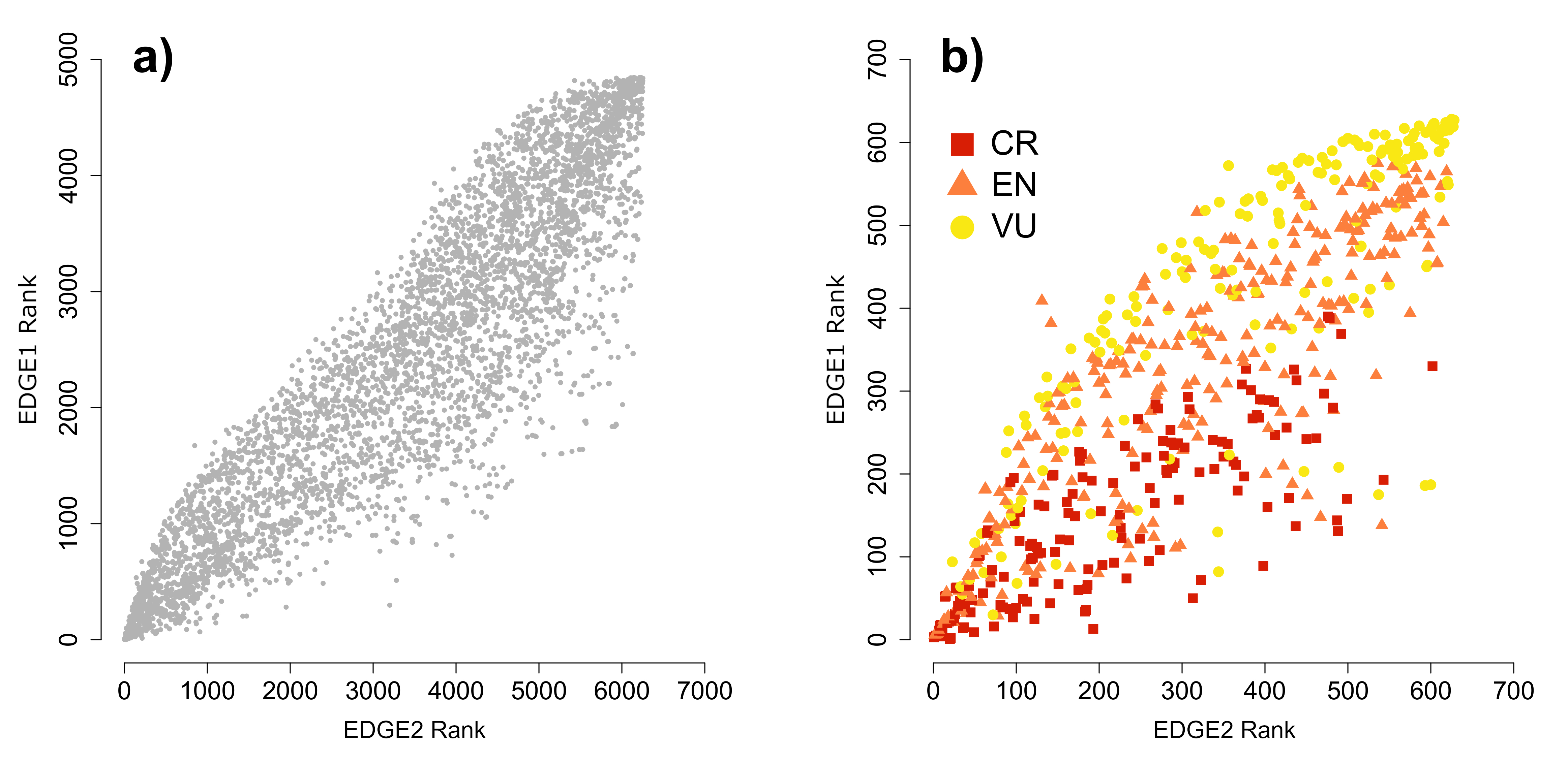

Supplement: S3 Fig — The EDGE2 ranks (x-axis) and EDGE1 ranks (y-axis) for (a) all mammals with data-sufficient and extant IUCN Red List assessments; and (b) all EDGE2 Species (above-median EDGE2 for 95% of iterations and threatened on IUCN Red List; see “EDGE2 Framework”). More details in S2 Text. The data underlying this Figure can be found in S2 Data. (TIF) [file pbio.3001991.s007.tif]

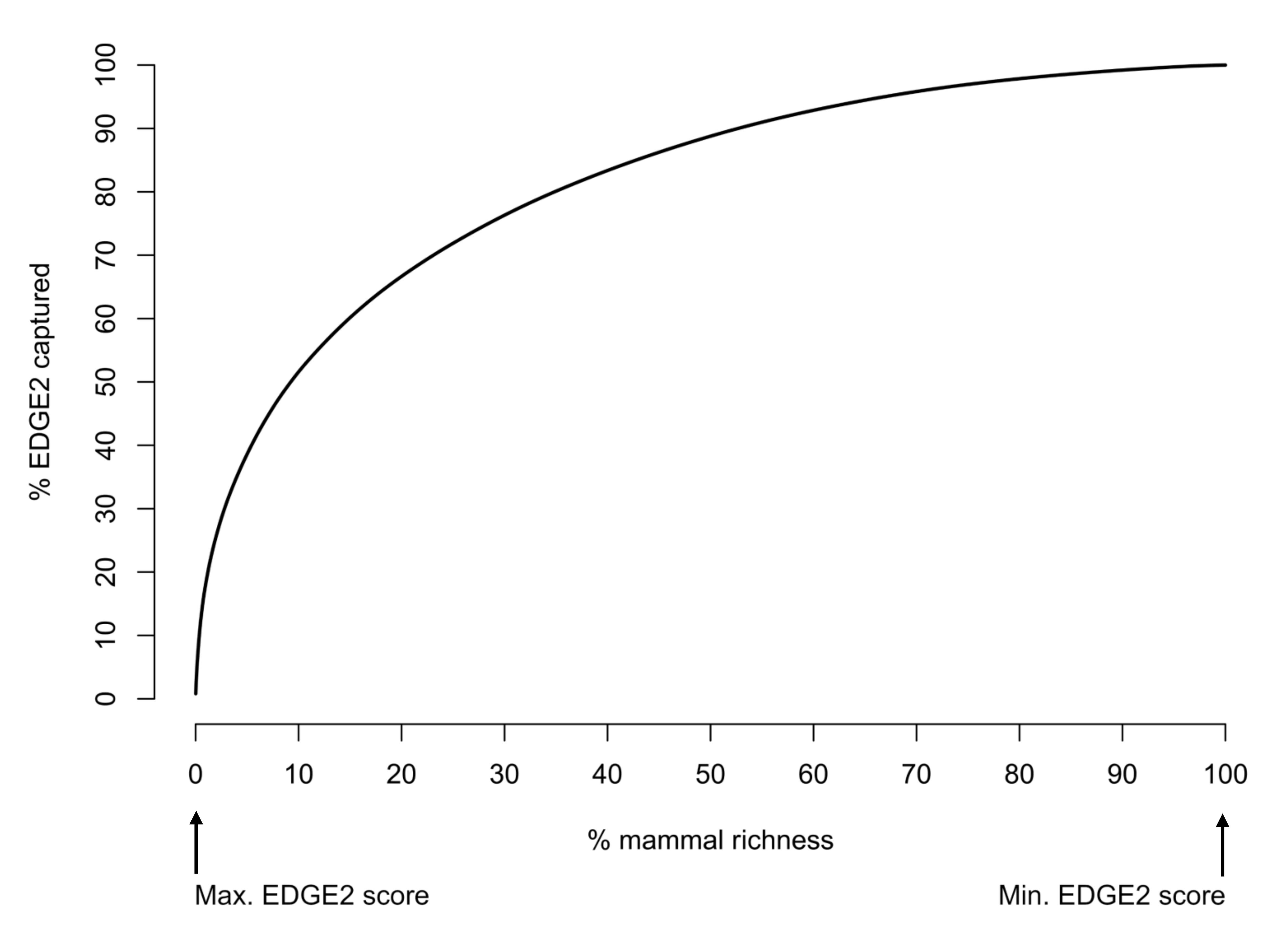

Supplement: S4 Fig — Cumulative percentage of total EDGE2 captured by increasing the number of species captured, from the mammal species with the highest EDGE2 score to the lowest. EDGE2 scores are cumulatively summed from the highest score to the lowest until all EDGE2 scores are captured. The data underlying this Figure can be found in S2 Data. (TIF) [file pbio.3001991.s008.tif]

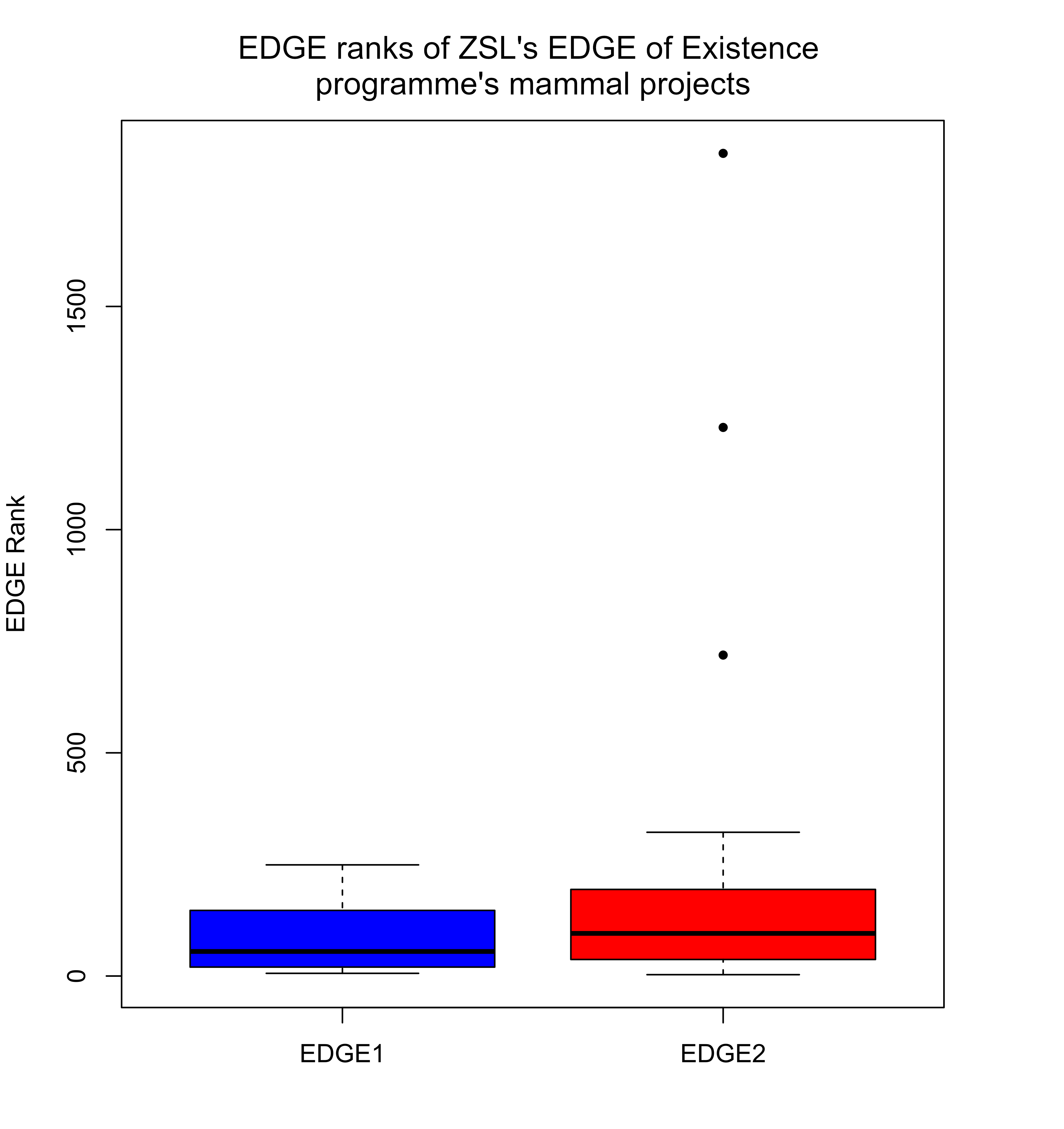

Supplement: S6 Fig — The EDGE1 and EDGE2 ranks of mammal species for which the ZSL’s EDGE of Existence programme has supported conservation projects. The data underlying this Figure can be found in S2 Data. EDGE, Evolutionarily Distinct and Globally Endangered; ZSL, Zoological Society of London. (TIF) [file pbio.3001991.s010.tif]
